# Systematic review of Janus kinases inhibitors for rheumatoid arthritis: methodology, reporting, and quality of evidence evaluation

**DOI:** 10.3389/fphar.2024.1459511

**Published:** 2024-09-25

**Authors:** Xiaolan Shen, Xiaoman Liu, Xiang Guo, Xiaoqiang Hou, Huiliang Huang, Zhitao Feng

**Affiliations:** ^1^ College of Medicine and Health Sciences, China Three Gorges University, Yichang, China; ^2^ The First College of Clinical Medical Sciences, Institute of Rheumatology, China Three Gorges University, Yichang, China; ^3^ The Second Clinical Medical School, Guangzhou University of Chinese Medicine, Guangzhou, China; ^4^ The Second People’s Hospital of Yichang, The Second Clinical Hospital of Three Gorges University, Yichang, Hubei, China

**Keywords:** JAK inhibitors, rheumatoid arthritis, AMSTAR-2, PRIAMA 2020, GRADE system

## Abstract

**Objective:**

To evaluate the methodological, reporting and evidence quality of systematic reviews or meta-analyses of Janus kinases (JAK) inhibitors for the treatment of rheumatoid arthritis (RA).

**Methods:**

Our study systematically retrieved reviews from various databases, spanning from inception to June 2024. Two evaluators independently assessed the methodological, reporting, and evidence quality of each review using the AMSTAR-2 and PRIAMA2020 tools. The evidence quality was evaluated according to GRADE criteria. Six aspects were evaluated: publication year, study type, homogeneity, risk of publication bias, AMSTAR-2 methodology, and PRIAMA2020 reporting quality. Excel 2016 facilitated conversion of scores into radar plots.

**Results:**

Following stringent selection criteria, a total of 18 relevant studies were identified. The AMSTAR-2 scores ranged from 4 to 13 points, with five studies rated as low quality and the remaining 13 as critically low quality. All studies encompassed populations, interventions, controls, and outcome measures, demonstrating commendable integrity. However, there is room for improvement in study protocol development and registration, comprehensive search strategies, inclusion and exclusion criteria, conflict of interest disclosure, and discussion of heterogeneity. PRIAMA2020 assessments ranged from 14.5 to 21 points, with two studies scoring below 15 points due to increased bias risk from data transformation and sensitivity analysis. Notably, all reviews (100%) adhered to PRIAMA2020 guidelines for certain items but none met all criteria. GRADE evaluation included 446 outcome measures, with 158 of moderate, 156 of low, and 132 of very low quality, indicating JAK inhibitors is effective in improving RA. According to radar chart, the average rank score was 13.13. One study achieved a balanced score across all dimensions, while 11 exceeded the average, five showed significant differences in PRIAMA2020 scores, and four in AMSTAR two scores.

**Conclusion:**

Despite summarizing the efficacy and safety of JAK inhibitors in treating RA, the included studies exhibited poor methodological and reporting quality, along with low-quality evidence overall. Therefore, caution is warranted among decision-makers regarding the use of JAK inhibitors in RA treatment. Urgent requirements include high-quality, multicenter studies investigating JAK inhibitors for RA.

**Systematic Review registration:**

https://www.crd.york.ac.uk/PROSPERO, identifier 413415.

## 1 Introduction

Rheumatoid arthritis (RA) is the most general chronic autoimmune disease, which is characterized by symmetrical polyarthritis that may cause bone and cartilage destruction, affecting the synovial tissue of the facet joints of the hands and feet, thereby causing tenderness, swelling and destruction of the joints (Mengdi et al., 2024; Díaz-González and Hernández-Hernández, 2023; Szekanecz et al., 2024). The current prevalence of RA is about 0.5%–1% (Brown et al., 2024). In addition, it was found that patients diagnosed with RA who had not received effective treatment within 2 years will have a 10-year disability rate of 50% according to the previous studies (Xiang et al., 2023). At present, the drugs for the treatment of RA are mainly nonsteroidal anti-inflammatory drugs (NSAIDs), glucocorticoids, biological and targeted small-molecule drugs, etc (Singh, 2022; Guo et al., 2020). Those played an important role in reducing pain, joint tenderness and improving the function of joints (Xu et al., 2019; Radu and Bungau, 2021). However, a considerable number of patients had no response to the current treatment drugs, especially for joint pain and swelling, its application alone cannot fully achieve the effect of delaying the disease process and joint destruction (Guo et al., 2021; Zhu et al., 2020). Therefore, it is considered necessary to find a new or complementary treatment. According to the preceding research, we discovered that JAK inhibitors have been continuously developed for the treatment of RA and other autoimmune diseases in recent years (Szekanecz et al., 2024; Bonelli et al., 2024).

Janus kinase (JAK) inhibitors are small molecular biologic agents belonging to the intracellular tyrosine kinase family, which play a pivotal role in the signaling of many cytokine receptors, mediating inflammation and autoimmune diseases (Roskoski, 2022; Ipek et al., 2023). In recent years, JAK inhibitors have been widely used to treat RA and proved to have an obvious curative effect (Smolen et al., 2020; Langbour et al., 2023). However, the efficacy and safety of the clinical use of JAK inhibitors need to be further verified (Clarke et al., 2021; Cohen and Reddy, 2023). In recent years, several articles have reported on the systematic reviews and meta-analyses of JAK inhibitors in the treatment of RA (20, 21). However, JAK inhibitors still face a series of deficiencies such as rigorous scientific design, widely varied outcomes as well as lack of clinical sample size.

Therefore, the objective of this study is to comprehensively assess the methodological and evidence quality of JAK inhibitors in the treatment of rheumatoid arthritis, with the aim of providing valuable evidence for clinical decision-making. The assessment will be carried out using the Aid of the Measurement Tool for Evaluation of Systematic Reviews (AMSTAR-2), the Preferred Reporting Item for Systematic Reviews and Meta-Analyses 2020 (PRIAMA 2020), and the Grading of Evaluation (GRADE) system for assessment (Shea et al., 2017; Page et al., 2021; Guyatt et al., 2011). It is expected to provide valuable information for the implementation in the field of JAK inhibitors for RA through this study.

## 2 Material and methods

### 2.1 Search strategy

This study conducted a comprehensive search of the PubMed, Web of Science, Scopus, EMBASE, Livivo, China Scientific Journal Database (VIP), China National Knowledge Infrastructure (CNKI), and Wan Fang Database from inception to June 2024. Additionally, searches were also performed in PubMed Central and Open Grey to acquire gray literature. There were no language or publication time limitations. The search terms included (“rheumatoid arthritis” OR “RA”) AND (“Janus Kinase inhibitor” OR “JAK inhibitor” OR “Ruxolitinib” OR “Baricitinib” OR “Tofacitinib” OR “Fedratinib” OR “Momelotinib” OR “Pacritinib” OR “Fligotinib” OR “Upadacitinib” OR “Itacitinib” OR “Decernotinib” OR “Peficitinib” OR Abrocitinb” OR Ritlecitnibi) AND (“meta-analyses” or “systematic review”). Search strategies in various databases are shown in Supplementary Tables 1, 2.

### 2.2 Inclusion and exclusion criteria

The evaluation criteria are as follows: (i) Randomized controlled trial (RCT) or Non-randomized controlled trial; (ii) patients diagnosed with rheumatoid arthritis according to the 1987 American College of Rheumatology guidelines, aged 18–60 years; (iii) RA patients treated with JAK inhibitors in combination with leflunomide (LEF), methotrexate (MTX), hydroxychloroquine or other DMARDs, and controls treated with LEF, MTX, hydroxychloroquine, or other DMARDs alone.

The following articles will be excluded: (i) Reviews not related to rheumatoid arthritis or other complications of rheumatoid arthritis; (ii) Outside of a system review or meta-analysis or network meta-analysis; (iii) Evaluation of treatment without JAK inhibitors; (iv) Repeated comments; (v) Review of data, results and full text with obvious defects.

### 2.3 Literature screening and data extraction

The retrieved literature was imported into the literature management software Endnote X7 to remove duplicates. The two evaluators (Xiaolan Shen and Xiaoman Liu) completed literature screening, data sorting and scoring utilizing the evaluation tool independently. If the evaluation results were inconsistent, the two evaluators made a decision through consultation; if there was still exist a disagreement, a third person would be consulted (Zhitao Feng). The review that met the criteria was extracted: journals, publication time, authors, number of case, intervention methods, treatment groups, control groups, and outcome indicators.

### 2.4 Report quality and methodological evaluation

The methodological, reporting, and evidence quality of all included systematic reviews and meta-analyses were assessed using the AMSTAR-2, PRISMA 2020, and GRADE tools, respectively. (Shea et al., 2017; Page et al., 2021; Guyatt et al., 2011).

The AMSTAR-2 assessment consists of 16 items, items 2, 4, 7, 9, 11, 13, and 15 were identified as critical according to the guidelines. Items that fully meet the evaluation criteria are rated as “Yes”; those partially meeting the criteria are rated as “Partial Yes”; and items with no relevant information reported in the systematic review are rated as “No.” At the end of the assessment, reviews are categorized into four levels: high (one non-critical flaw), moderate (more than one non-critical flaw), low (one critical flaw, with or without non-critical flaws), and critically low (more than one critical flaw, with or without non-critical flaws) (Shea et al., 2017). The rationale of items of AMSTAR-2 is shown in Supplementary Table 3.

PRIAMA 2020 statement is designed to assess the completeness of information reporting in systematic reviews. It comprises a 27-item checklist organized into seven sections, each detailing reporting recommendations and providing examples for each item. The evaluation criteria include three categories: “Yes” (completely satisfies the criterion), “Partial Yes” (partially satisfies the criterion), and “No” (does not satisfy the criterion). Points are assigned as 1, 0.5, or 0, respectively, resulting in a total score ranging from 0 to 27 (23). The PRIAMA 2020 checklists are shown in Supplementary Table 4.

In this study, the GRADE tool was mainly used to assess the quality of evidence across all included reviews. The evidence was categorized as high (the true effect is likely very close to the estimated effect), moderate (the true effect is probably close to the estimated effect, but there is a possibility of a significant difference), low (the true effect may be considerably different from the estimate), and very low (the true effect is likely to be substantially different from the estimate). Additionally, five factors that could lead to downgrading the quality of evidence were considered: risk of bias, publication bias, imprecision, inconsistency, and indirectness. Each outcome measure was assessed, with downgrading factors rated as “not serious” or “serious” (resulting in a one-level downgrade, −1). Studies were also upgraded based on effect size and dose-response, with each upgrade resulting in a one-level increase (+1). The GRADE System tool checklists are shown in Supplementary Table 5.

The evaluation of reporting, methodological, and evidence quality evaluations were independently conducted by two appraisers using the same criteria to assess the quality of the included reviews. Any disagreements were resolved through consensus discussions, and if an agreement could not be reached, a third examiner was consulted to finalize the decision.

### 2.5 Create a radar map

Extract the publication year, homogeneity level, research type, publication bias test, AMSTAR-2 and PRIAMA2020 evaluations from various system evaluations/meta analyses. Given the importance of timeliness in system evaluations, literature published more recently holds greater relevance. Therefore, literature with a publication year closer to the current date is assigned a higher rank. The distinction between high and low homogeneity in literature is determined by specific criteria: a Q test result of *p* ≥ 0.1 indicates high homogeneity, whereas a heterogeneity test result of *I* ^2^ ≤ 50% signifies low homogeneity. In terms of research types, randomized controlled trials (RCTs) are considered to be of high rank due to their rigorous methodology, while quasi-randomized control trials (qRCTs) receive a lower rank due to their less stringent design. Furthermore, literature that has undergone publication bias testing is categorized as high rank, emphasizing its credibility, whereas literature lacking such testing is deemed low rank. Upon evaluating each piece of literature across various criteria, the cumulative rank is represented in a radar chart, providing a visual assessment of the literature’s quality. The literature is then sorted by rank, with a higher rank reflecting superior quality and evaluation results for the item.

Extract the publication year, homogeneity level, research type, publication bias test results, and AMSTAR-2 and PRISMA 2020 evaluations from various systematic reviews and meta-analyses. Given the importance of timeliness, more recent literature is ranked higher. Homogeneity is classified based on specific criteria: a Q test result of *p* ≥ 0.1 indicates high homogeneity, while an *I* ^2^ ≤ 50% indicates low heterogeneity. Types of research are ranked based on methodological rigor, with randomized controlled trials (RCTs) being considered superior to quasi-randomized controlled trials (qRCTs) due to the latter’s less stringent design. Literature that includes a publication bias test is given higher credibility, while those lacking such testing are rated lower. After evaluating each study according to these criteria, the cumulative rank is presented in a radar chart, providing a clear assessment of the literature’s quality. Finally, the literature is sorted by rank, with higher ranks indicating superior quality and evaluation outcomes.

## 3 Results

### 3.1 Literature screening process and basic information

**FIGURE 1:**
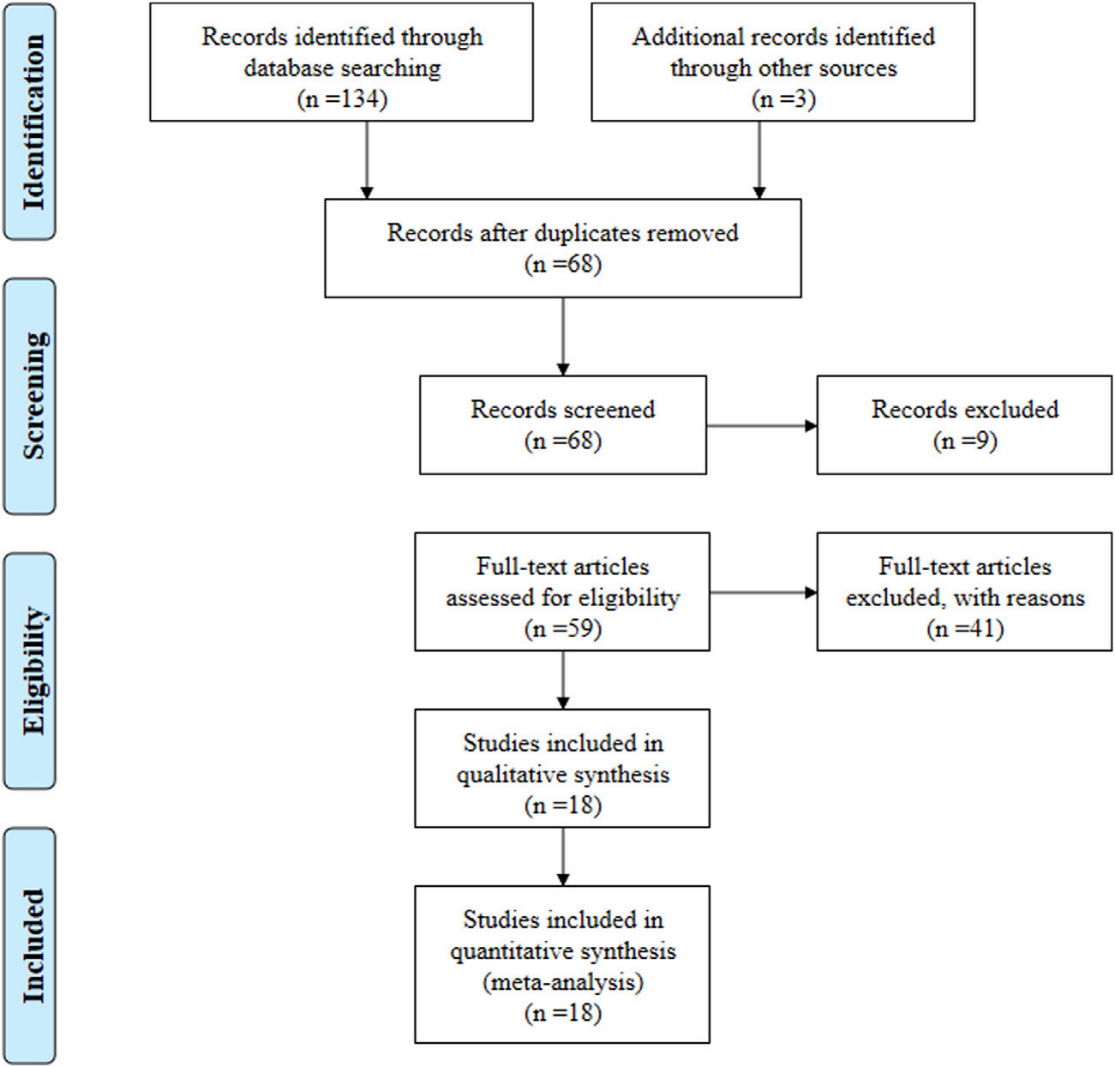
Flow diagram of the included study screening procedure.

According to the search strategy, a total of 137 relevant articles were initially obtained. Aafter step-by-step screening, 18 systematic reviews from 2013 to 2023 (Liu et al., 2022; Tóth et al., 2022; Qian et al., 2022; Xin et al., 2020; Shiqin et al., 2019; Ya et al., 2015; Yuan and Fang, 2018; Chunyan et al., 2018; Lan et al., 2020; Kunwar et al., 2018; Wang et al., 2022; Yin et al., 2021; He et al., 2013; Wang et al., 2020; Sung and Lee, 2022; Sung and Lee, 2023; Kawalec et al., 2013; Zhang et al., 2014) were finally included, comprising of 11 in English and seven in Chinese. Figure 1. The characteristics of the included studies are summarized in Table 1.

**TABLE 1:** Basic information and characteristic of all included reviews.

Study	Years	RCT/Patients	Treatment intervention	Control intervention	Quality assessment	Conclusion
Zhao	2022	5/3544	Filgotinib (50 mg/100 mg/200mg, qd)	placebo/MTX	Cochrane	Filgotinib is effective for RA with mild side effects, and it may be a new strategy for RA. Due to the limited number of literature included in this study, which needs support from future studies
Zhang	2018	9/3742	Tofacitinib (5/10 mg,bid,12w)+MTX	placebo	Cochrane	Tofacitinib have considerable effect but with more mild adverse events in the treatment of rheumatoid arthritis
Qin	2018	6/3546	Baricitinib (2mg/4 mg,bid)+MTX/DMARDs	placebo + MTX/DMARDs	Cochrane	Baricitinib is significantly effective in the treatment of rheumatoid arthritis but increases the incident of infection and herpes zoster
Liu	2020	5/1773	Peficitinib	placebo + MTX/DMARDs	Cochrane + Jadad	For the treatment of RA, 100 mg or 150 mg peficitinib once per day is superior to placebo in terms of ACR20, ACR50 and ACR70, DAS28- ESR<2.6, DAS28- CRP<2.6; the adverse events are mild and tolerable and it may be a new treatment option for RA.
Yao	2020	7/4208	Upadacitinib + DMARDs	placebo + adalimumab/MTX	Cochrane	Upadacitinib is beneficial to the improvement of the condition of RA patients with high safety and tolerability, but which still needs to be verified by large-sample, multi-left clinical trials
Liu	2015	8/2909	Tofacitinib (5mg/10 mg,bid)	placebo	Cochrane	Tofacitinib is effective for the treatment of RA and equal to placebo in safety. It is a safe and effective therapy for RA. However, as the short duration and the limited sources may somewhat affect results, more high-guality double-blind RCTs may be required for further assessment of the effects
Li	2019	8/2738	Filgotinib + Upadacitinib + MTX/DMARDs	placebo	Cochrane	JAK-1 inhibitors Upadacitinib and Filgotinib can improve the effect indexes of ACR20, ACR50 and ACR70 and the proportion of patients with DAS28 < 3.2 of rheumatoid arthritis patients; it can not increase the incidence of SAE, severe infection, herpes zoster, liver injury, but can increase the risk of AE and infection
Kunwar	2018	5/2458	Baricitinib	placebo	Cochrane	Baricitinib is effective in treatment of RA, and did not appear to have significant safety concerns during the first 6 months of treatment
Lilla	2022	33/24135	JAK inhibitors	placebo + MTX/DMARDs	Cochrane	Analgesic effect determined using the visual analogue scale and American College of Rheumatology (ACR) 20/50/70 response rates was significantly greater in the JAK group in all comparisons, and no significant difference regarding safety could be explored. This meta-analysis gives a comprehensive overview of JAK inhibitors and provides evidence for their superiority in improving PROs and disease activity indices in RA.
Liu	2022	3/2290	JAK inhibitors + MTX	placebo	Cochrane	JAKi combined with MTX demonstrated superiority to JAKi monotherapy in terms of ACR responses, low disease activity and remission achievement. The two regimens presented comparable physical functioning measured by HAQ-DI improvement and similar tolerability, except for high risks of TEAEs and AEs leading to study discontinuation in combination therapy
Yin	2021	28/14500	JAK inhibitors	placebo/DMARDs	Jadad	Jakinibs are efficacious and well tolerated in RA patients up to 24 weeks, although they are associated with an increased risk of infectious complications
Sung	2021	19/3442	JAK inhibitors + TNF inhibitors	placebo	Cochrane	There were higher placebo and less nocebo effects of JAK vs non-TNF inhibitors in RA patients with an insufficient response to TNF inhibitors, and the greater the placebo response, the weaker the nocebo response and the greater the efficacy
Sung	2021	5/1422	JAK inhibitors	placebo	Cochrane	Placebo and nocebo effects have signif icant consequences for the nature and practice of RCTs. In addition to efficacy, safety, and tolerability, the potential of a drug to be correlated with a placebo or nocebo response is an important feature of medicines and may predict treatment success
Zhang	2013	10/4929	Tofacitinib + MTX/DMARDs	placebo + MTX/DMARDs	Cochrane	For patients with an inadequate response to DMARD, taking tofacitinib alone or together with non-biologic DMARDs was associated with more favorable remission in the signs and symptoms of RA than adalimumab or placebo. Also, the study indicated tofacitinib monotherapy was safer than place bo with respect to reported sAEs, but not oAEs. There was no evidence of significant difference in the safety of tofacitinib compared with placebo when both were combined with back ground therapy. However, based on the grading of the evi dence using the GRADE approach, the quality of evidence for tofacitinib as therapy for RA patients who have had an inad equate response to at least one DMARD is exceedingly low
Wang	2020	20/8982	Tofacitinib + Baricitinib + Upadacitinib	placebo	Cochrane	Tofacitinib, baricitinib, and upadacitinib significantly improve RA control. Head-to-head Janus activated kinase inhibitor clinical trials are needed to further inform decision making
Wang	2022	37/15174	JAK inhibitors	placebo	Cochrane	JAKinibs are effective at reducing RA signs and symptoms of RA, and improve health related quality of life, but the safety concerns should be paid attention. Increased risk of infections and AE were observed in baricitinib and upadacitinib, whereas only baricitinib statistically increased the risk of HZ. However, this study was limited by its short duration (less than 24 weeks). Further trials are necessary to assess long-term safety, especially for decernotinib, peficitinib, and fligotinib
He	2013	8/3791	Tofacitinib + MTX	placebo + MTX	Cochrane	Tofacitinib is efficacious and well tolerated in patients with MTX-resistant RA up to a period of 24 weeks. However, haematological, liver function tests and lipoproteins should be monitored. Long-term efficacy and pharmacovigilance studies are recommended
Kawalec	2013	8/-	Tofacitinib + MTX/DMARDs	placebo + MTX/DMARDs	Cochrane	Tofacitinib monotherapy or with background methotrexate provides ear ly statistically significant and clinically important improve ment in rheumatoid arthritis symptoms and has an acceptable safety profile comparable to that of placebo. The results of the present meta-analysis show that the frequency of serious adverse events was not increased after tofacitinib treatment. In addition, tofacitinib might provide an effective treatment option compared to intravenous or subcutaneous biological DMARDs, as suggested by the result of the comparison made regarding tofacitinib vs adalimumab ACR50 response rate

### 3.2 Methodological quality assessment

The AMSTAR-2 assessment included seven key evaluation areas: items 2, 4, 7, 9, 11, 13, and 15. Only one article met the second criterion; one article failed to meet the fourth criterion; five articles did not meet the seventh criterion; one article failed the ninth criterion; two articles did not meet the 11th criterion; three articles fell short of the 13th criterion, and seven articles did not meet the 15th criterion. The 18 studies included in AMSTAR-2 exhibited scores ranging from four to 13. Of these, 13 were classified as critically low quality, while five were categorized as low quality. None of them met the criteria for medium or high quality Table 2.

**TABLE 2:** The evaluation results of the included reviews of AMSTAR-2.

	1	2	3	4	5	6	7	8	9	10	11	12	13	14	15	16	Score	Quality
Zhao	Y	N	N	PY	Y	Y	Y	Y	Y	N	Y	N	N	Y	Y	N	9.5	Critically Low
Zhang	Y	N	N	PY	Y	Y	Y	Y	Y	N	Y	Y	Y	Y	Y	N	11.5	Critically Low
Qin	Y	N	N	PY	Y	Y	N	Y	Y	N	Y	Y	Y	Y	Y	N	10.5	Critically Low
Liu	Y	N	N	PY	Y	Y	Y	Y	Y	N	Y	Y	Y	N	N	N	9.5	Critically Low
Yao	Y	N	N	Y	Y	Y	PY	Y	Y	N	Y	Y	N	N	N	N	8.5	Critically Low
Liu	Y	N	N	N	Y	Y	N	N	N	N	Y	N	N	N	N	N	10.5	Critically Low
Li	Y	N	N	PY	Y	Y	N	Y	Y	N	Y	Y	Y	Y	Y	N	10.5	Critically Low
Kunwar	Y	N	N	PY	Y	Y	PY	Y	Y	N	Y	Y	Y	Y	N	N	10	Critically Low
Lilla	Y	N	N	PY	Y	Y	PY	Y	N	N	Y	Y	Y	N	N	Y	9	Critically Low
Liu	Y	Y	N	Y	Y	Y	Y	Y	Y	N	Y	Y	Y	N	N	Y	12	Low
Yin	Y	N	N	Y	Y	Y	PY	Y	N	N	Y	Y	Y	Y	N	Y	10.5	Critically Low
Sung	Y	N	N	PY	N	N	N	N	PY	N	N	Y	N	N	N	Y	4	Critically Low
Sung	Y	N	N	PY	N	N	N	N	Y	N	N	Y	Y	Y	Y	Y	7.5	Critically Low
Zhang	Y	N	N	PY	Y	Y	Y	Y	Y	N	Y	Y	Y	Y	Y	N	11.5	Critically Low
Wang	Y	N	N	Y	Y	Y	Y	Y	Y	N	Y	Y	Y	Y	Y	Y	13	Low
Wang	Y	N	N	Y	Y	Y	Y	Y	Y	N	Y	Y	Y	Y	Y	Y	13	Low
He	Y	N	N	Y	Y	Y	Y	Y	Y	N	Y	Y	Y	Y	Y	Y	13	Low
Kawalec	Y	N	N	Y	Y	Y	Y	Y	Y	N	Y	N	Y	Y	N	Y	11	Critically Low

Y, YES; N, NO; PY, Partial YES.

All eighteen reviews (100%) adhered to the PICO principle,covering population, intervention, control group, and outcome. Thirteen studies (72%) explicitly stated their inclusion and exclusion criteria. In addition, eleven studies (61%) employed a rigorous method to assess the risk of bias in the randomized controlled trials, considering their potential impacts on the meta-analyses results or evidence integration. These studies used appropriate statistical models, predicted the likely incidence of review outcomes, investigated sources of heterogeneity, and discussed their influence on the findings. Furthermore, sixteen studies (89%) involved multiple investigators who independently conducted screening and evaluation. Finally, eine studies (50%) demonstrated no conflicts of interest. The methodological quality evaluation results of the included reviews of AMSTAR-2 in Table 3.

**TABLE 3:** Methodological quality of all included reviews by AMSTAR-2 assessment.

Item	YES		Partial YES		NO	
	Frequency	proportion (%)	Frequency	proportion (%)	Frequency	proportion (%)
1	18.00	100.00	0.00	0.00	0.00	0.00
2	1.00	5.56	0.00	0.00	17.00	94.44
3	0.00	0.00	0.00	0.00	18.00	100.00
4	7.00	38.89	10.00	55.56	1.00	5.56
5	16.00	88.89	0.00	0.00	2.00	11.11
6	16.00	88.89	0.00	0.00	2.00	11.11
7	9.00	50.00	4.00	22.22	5.00	27.78
8	15.00	83.33	0.00	0.00	3.00	16.67
9	14.00	77.78	1.00	5.56	3.00	16.67
10	0.00	0.00	0.00	0.00	18.00	100.00
11	16.00	88.89	0.00	0.00	2.00	11.11
12	15.00	83.33	0.00	0.00	3.00	16.67
13	14.00	77.78	0.00	0.00	4.00	22.22
14	16.00	88.89	0.00	0.00	2.00	11.11
15	9.00	50.00	0.00	0.00	9.00	50.00
16	9.00	50.00	0.00	0.00	9.00	50.00

Thirteen reviews were classified as critically low quality due to the presence of multiple key flaws, while the remaining five reviews were categorized as low quality based on the AMSTAR-2 evaluation criteria.

### 3.3 Evaluation of report quality

The PRIAMA2020 scores ranged from 14.5 to 21, with four of the 18 studies analyzed scoring between 21 and 27, fourteen scoring between 15 and 21, and only two studies receiving scores between 0 and 15 Table 4. All the reviews (100%) conform to the specification of item 1, 3, 4, 15, 16a, and 23 (a-d) in the PRIAMA2020 structure. However, none of the reviews (0%) conform to the specification of items 24 (b-c). The title, background, theoretical basis, purpose, evaluation method, outcome indicators, individual study results, and inter-study bias risk were adequately reported. Nevertheless, there was insufficient reporting on the causes of bias risk as well as on methods such as combining effect size, data transformation and conducting sensitivity analysis to ensure study stability. Additionally, details regarding study registration, protocol adherence along with public information were rarely provided. Partial reporting was observed for other components of the PRIAMA2020 statement as depicted in Table 5.

**TABLE 4:** The evaluation results of the included reviews of PRIAMA 2020-2020.

	Zhao	Zhang	Qin	Liu	Yao	Liu	Li	Kunwar	Lilla	Liu	Yin	Sung	Sung	Zhang	Wang	Wang	He	Kawalec
1	Y	Y	Y	Y	Y	Y	Y	Y	Y	Y	Y	Y	Y	Y	Y	Y	Y	Y
2	PY	PY	PY	PY	PY	PY	PY	PY	PY	PY	PY	PY	PY	PY	PY	PY	PY	PY
3	Y	Y	Y	Y	Y	Y	Y	Y	Y	Y	Y	Y	Y	Y	Y	Y	Y	Y
4	Y	Y	Y	Y	Y	Y	Y	Y	Y	Y	Y	Y	Y	Y	Y	Y	Y	Y
5	PY	Y	PY	PY	PY	PY	PY	PY	PY	Y	Y	PY	PY	Y	Y	PY	PY	Y
6	PY	PY	PY	PY	PY	PY	PY	PY	PY	PY	PY	PY	PY	PY	PY	PY	PY	PY
7	Y	Y	Y	Y	Y	Y	Y	Y	Y	Y	Y	PY	PY	PY	Y	PY	PY	Y
8	Y	Y	Y	Y	Y	Y	Y	Y	Y	Y	Y	N	N	Y	Y	Y	Y	Y
9	Y	Y	Y	Y	Y	Y	Y	Y	Y	Y	Y	N	N	Y	PY	Y	Y	Y
10a	PY	PY	PY	PY	PY	PY	PY	PY	PY	PY	PY	PY	PY	PY	PY	PY	PY	PY
10b	PY	PY	PY	PY	PY	PY	PY	PY	PY	PY	PY	PY	PY	PY	Y	Y	PY	PY
11	Y	Y	Y	Y	Y	Y	PY	PY	Y	Y	PY	PY	PY	PY	PY	PY	PY	Y
12	Y	Y	Y	Y	Y	Y	Y	PY	PY	Y	PY	Y	Y	Y	PY	Y	Y	Y
13a	PY	PY	PY	PY	PY	PY	PY	PY	PY	PY	PY	PY	PY	PY	PY	PY	PY	PY
13b	PY	N	N	N	N	Y	N	N	Y	N	N	N	N	Y	Y	Y	N	Y
13c	Y	Y	Y	Y	Y	Y	Y	Y	Y	Y	Y	Y	Y	Y	PY	PY	PY	Y
13d	PY	PY	PY	PY	PY	PY	PY	PY	PY	PY	PY	PY	PY	PY	PY	PY	PY	PY
13e	Y	Y	Y	Y	Y	Y	Y	Y	Y	Y	Y	N	N	Y	Y	PY	Y	PY
13f	Y	Y	Y	Y	Y	Y	Y	PY	PY	PY	PY	PY	PY	PY	PY	PY	Y	Y
14	PY	Y	Y	Y	PY	Y	PY	PY	Y	PY	PY	PY	Y	PY	Y	Y	PY	Y
15	Y	Y	Y	Y	Y	Y	Y	Y	Y	Y	Y	Y	Y	Y	Y	Y	Y	Y
16a	Y	Y	Y	Y	Y	Y	Y	Y	Y	Y	Y	N	N	Y	Y	Y	Y	Y
16b	Y	Y	Y	Y	Y	Y	Y	Y	PY	Y	Y	Y	Y	Y	Y	Y	Y	Y

Y, YES; N, NO; PY, Partial YES.

**TABLE 5:** Reporting quality of all included reviews by PRIAMA 2020-2020 system.

Section	Item	YES		Partial YES		NO	
		Frequency	Proportion (%)	Frequency	Proportion (%)	Frequency	Proportion (%)
Title	1	18.00	100.00	0.00	0.00	0.00	0.00
Abstract	2	0.00	0.00	18.00	100.00	0.00	0.00
Introduction	3	18.00	100.00	0.00	0.00	0.00	0.00
	4	18.00	100.00	0.00	0.00	0.00	0.00
Methods	5	6.00	33.33	0.00	0.00	12.00	66.67
	6	0.00	0.00	18.00	100.00	0.00	0.00
	7	13.00	72.22	0.00	0.00	5.00	27.78
	8	16.00	88.89	0.00	0.00	2.00	11.11
	9	15.00	83.33	1.00	5.56	2.00	11.11
	10a	0.00	0.00	18.00	100.00	0.00	0.00
	10b	2.00	11.11	16.00	88.89	0.00	0.00
	11	9.00	50.00	9.00	50.00	0.00	0.00
	12	14.00	77.78	4.00	22.22	0.00	0.00
	13a	0.00	0.00	18.00	100.00	0.00	0.00
	13b	6.00	33.33	1.00	5.56	11.00	61.11
	13c	15.00	83.33	3.00	16.67	0.00	0.00
	13d	0.00	0.00	18.00	100.00	0.00	0.00
	13e	14.00	77.78	2.00	11.11	2.00	11.11
	13f	9.00	50.00	9.00	50.00	0.00	0.00
	14	9.00	50.00	9.00	50.00	0.00	0.00
	15	18.00	100.00	0.00	0.00	0.00	0.00
Results	16a	16.00	88.89	0.00	0.00	2.00	11.11
	16b	17.00	94.44	1.00	5.56	0.00	0.00
	17	14.00	77.78	2.00	11.11	2.00	11.11
	18	8.00	44.44	10.00	55.56	0.00	0.00
	19	10.00	55.56	8.00	44.44	0.00	0.00
	20a	5.00	27.78	13.00	72.22	0.00	0.00
	20b	9.00	50.00	9.00	50.00	0.00	0.00
	20c	2.00	11.11	14.00	77.78	2.00	11.11
	20d	7.00	38.89	5.00	27.78	6.00	33.33
	21	4.00	22.22	14.00	77.78	0.00	0.00
	22	11.00	61.11	7.00	38.89	0.00	0.00
Discussion	23a	18.00	100.00	0.00	0.00	0.00	0.00
	23b	18.00	100.00	0.00	0.00	0.00	0.00
	23c	18.00	100.00	0.00	0.00	0.00	0.00
	23d	18.00	100.00	0.00	0.00	0.00	0.00
Other information	24a	4.00	22.22	0.00	0.00	14.00	77.78

### 3.4 Evidence quality evaluation

The evidence quality of the 18 included studies was assessed, encompassing a total of 446 outcome indicators. Among these, 158 indicators (35.42%) were classified as medium quality, 156 indicators (34.98%) were categorized as low quality, and 132 indicators (29.60%) were deemed to have extremely low quality. The quality evidence of included reviews is shown in Supplementary Table 6.

### 3.5 Efficacy evaluation results reported in the systematic review

In comparison to placebo, treatment with Filgotinib 200 mg resulted in improved ACR50/70 index scores among patients, and the study was assessed as having moderate quality. Similarly, Baricitinib 2 mg demonstrated a medium-quality improvement in ACR50/70 index scores, while Upadacitinib 15 mg significantly improved ACR20/50/70 index scores with a medium-quality evaluation. Furthermore, in comparison to monotherapy with JAK inhibitors alone, the combination of Baricitinib and Filgotinib with MTX demonstrated significant efficacy and received a medium-quality assessment according to the GRADE scale. These findings provide evidence supporting the effectiveness and scientific rationale for selecting JAK inhibitors in RA treatment, Supplementary Table 5.

### 3.6 The safety evaluation results of the reports included in the systematic review

In the assessment of adverse reactions caused by JAK inhibitors, Filgotinib demonstrated a relatively low risk profile for severe adverse reactions, herpes zoster, and upper respiratory tract infection. This suggests that Filgotinib exhibits a comparatively favorable safety profile, with its evidence quality being evaluated as moderate. Baricitinib 4 mg also exhibited relatively low risks of adverse reactions and infection, with its evidence quality being assessed as moderate. Similarly, Upadacitinib 15 mg displayed relatively low risks of adverse reactions, severe adverse reactions, and infection while maintaining a moderate level of evidence quality. These findings suggest that different JAK inhibitors possess distinct advantages in terms of their side effect profiles, including adverse reactions, infections and herpes zoster. This indicates varying levels of safety among the different JAK inhibitors, Supplementary Table 5.

### 3.7 Radar chart for system evaluation

**FIGURE 2:**
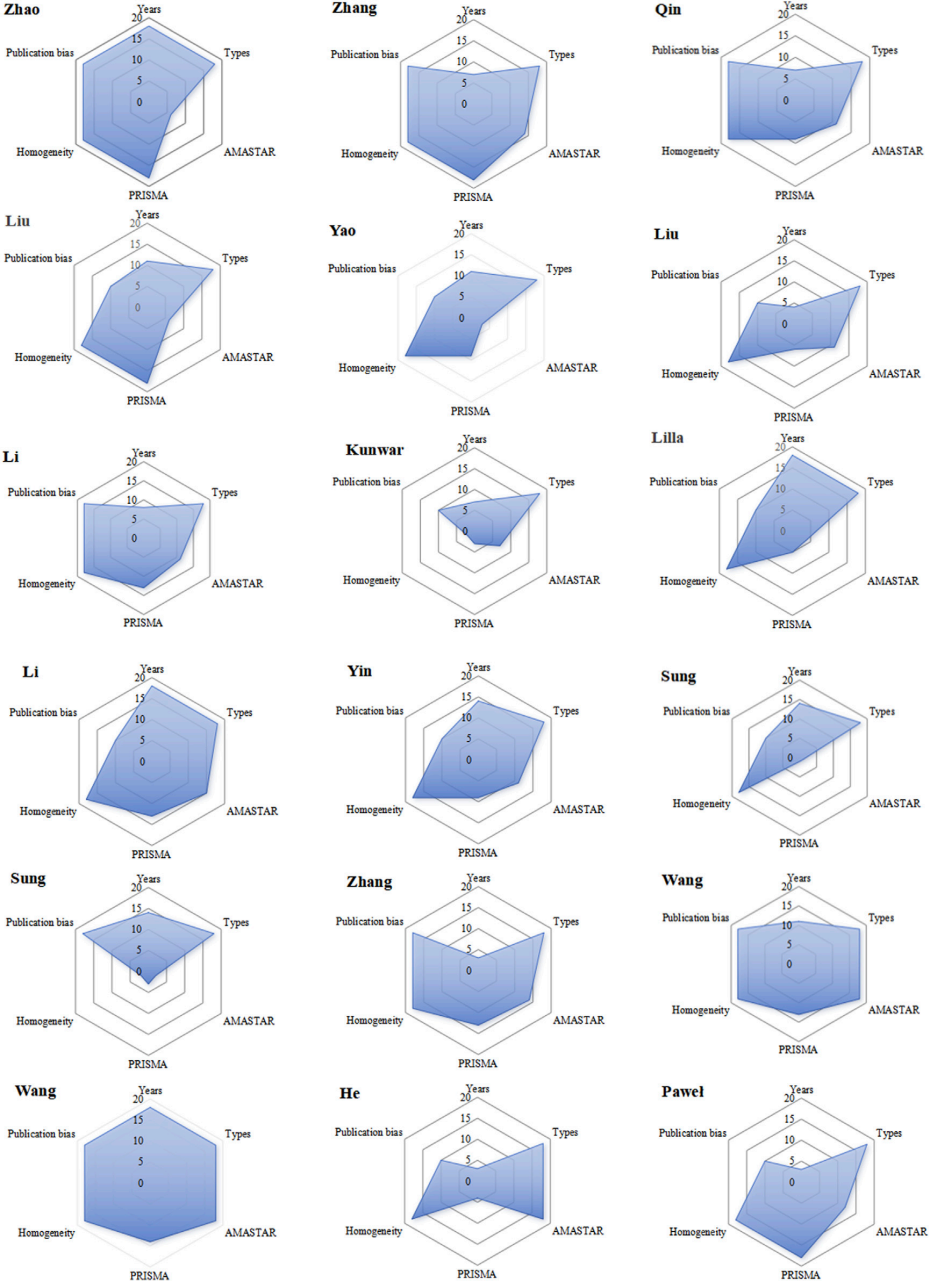
Radar chart for system evaluation.

The publication years range from 2013 to 2022, with more recent publications ranked higher. The highest rank of 18 is held by the 2022 publication, while the lowest rank of three belongs to one from 2013. In terms of study types, pure RCTs are given the highest rank, while those including qRCTs receive the lowest. All 18 publications are classified as RCTs, each with a rank of 18. Two publications were classified as having low homogeneity, while the remaining ones were considered highly homogeneous. Eight articles used funnel plots to test for publication bias, while the remaining ten did not, resulting in a rank of 10 for thosethat did not use funnel plots. In PRIAMA2020 scoring, the highest rank is associated with a score of 21 points and is given to publications with a ranking of 18; whereas the lowest rank receives a score of only14.5 points and is assigned to those with a ranking of 1. The sum ranks for six entries were ordered, with higher ranks indicating better quality literature. Radar charts based on these ranks were drawn, with 18 charts arranged chronologically. A larger radar chart area indicates higher quality and greater reference value of the literature, Figure 2. The average rank score of the included studies was 13. Combined with the intuitive radar map, Wang Faping’s study demonstrated the highest quality and more balanced scores in all dimensions, while Sumit Kunwar’s study had the lowest quality, with low scores in AMSTAR-2, PRIAMA2020 and homogeneity. Eleven studies scored above the average across all dimensions. It is worth noting that the PRISMA 2020 scores varied significantly in five studies, and the AMSTAR-2 scores varied significantly in four studies. Of particular concern are two studies by Sung, which had notably low AMSTAR-2 and PRISMA scores, directly impacting their average rank. However, most of the included studies were relatively recent, focused on RCTs, and exhibited low publication bias, contributing to their overall credibility.

## 4 Discussion

### 4.1 The methodological rigor requires enhancement

The purpose of this study was to appraise the methodological and reporting quality, along with the quality of evidence, from published systematic reviews/meta-analyses of the efficacy and safety of JAK inhibitors for RA. High-quality systematic reviews and meta-analyses are used to summarize related research and estimating the benefits and harms of interventions for patients and clinicians. Unfortunately, the AMSTAR two scale in this study showed that of the 18 included articles, five were evaluated as low quality and 13 were evaluated as critically low quality, indicating that the quality of the AMSTAR method needs to be improved.

According to the quality assessment results from the AMSTAR-2 methodology, several aspects require improvement, especially item 2 (pre-study protocol), item 3 (justification of study inclusion criteria), item 4 (comprehensive search strategy), item seven (exclusion criteria and justifications), item 10 (source of funding), item 14 (discussion of heterogeneity), item 15 (investigation of publication bias), and item 16 (reporting of conflicts of interest). The main reason for the overall low quality assessment is that 94.44% of the research did not adequately provide preliminary study protocols for the included systematic reviews. Additionally, none of the research explained the reasons for inclusion criteria, which may decrease consistency, increase selection bias risk, and reduce rigor. Furthermore, most included studies did not conduct comprehensive literature searches and did not provide detailed search strategies or explanations of their inclusion/exclusion criteria, which also increases the risk of publication bias. Moreover, although most studies assessed the risk of bias in their study design or execution process, they overlooked analyzing the potential causes and impacts of these biases on the results, resulting in decreased assessment quality. We strongly recommend that researchers register their studies on the international registry platform PROSPERO before conducting future research. However, it is noteworthy that despite these limitations, the included literature still provided detailed descriptions of study population characteristics, as well as intervention measures adopted in conjunction with control measures and outcome indicators, thereby enhancing the comprehensiveness and integrity of the articles. Additionally, by assessing result heterogeneity and bias risk, most studies also employed appropriate methods to partially mitigate the risks associated with selection and publication bias.

To enhance comprehensiveness and reduce publication bias, it is recommended to plan and conduct comprehensive searches using professional databases, journals, and grey literature. Authors have a responsibility to disclose funding sources and conflicts of interest to avoid biasing the study towards sponsored products, and should conclude their reports with convincing results.

### 4.2 The quality of literature reports need to be improved

The PRIAMA2020 assessment results indicated that four papers achieved scores ranging from 21 to 27, reflecting a more comprehensive research methodology. Furthermore, 13 papers scored between 15 and 21, suggesting some flaws in the research method. Only one paper scored below 15, highlighting serious deficiencies in the conducted research. These findings imply the need for improving the quality of the included literature.

The limitations contributing to these issues are that all literature sources lacked complete structured abstracts with missing data source information, detailed interventions, and comprehensive data analysis methods; thereby compromising study integrity. Additionally, a number of studies inadequately described essential methods required for presenting or synthesizing data which may introduce subjectivity and ambiguity while increasing selection bias. What’s more, most of the included studies lacked registration information, leading to inconsistencies before and after implementing the research protocol, which significantly undermined study credibility due to subjective inclusion/exclusion of data. Finally, certain studies had incomplete literature retrieval by only considering a few databases without accounting for gray literature or manual supplementary methods; this affected study integrity.

In order to improve the quality of research, we strongly appeal that researchers should strictly adhere to the PRIAMA2020 checklist, and editors should require authors to adhere to updated assessment tools in addition to AMSTAR-2, PRIAMA 2020, or GRADE before accepting manuscripts. Most importantly, clinical trials are crucial in systematic reviews, and researchers should undergo rigorous training in conducting clinical trials to ensure high-quality systematic reviews.

### 4.3 Effectiveness of JAK inhibitors

In terms of clinical efficacy, firstly, JAK inhibitors demonstrated significant improvements in ACR20/50/70 indicators compared to placebo, indicating their advantageous role in treating RA. Filgotinib exhibited superior efficacy at 200 mg with medium-quality evaluations for ACR50/70 indicators. Baricitinib at 2mg and 4 mg showed certain advantages with medium-quality evaluations for ACR50/70 indicators. Upadacitinib treatment at 15 mg resulted in significantly improved ACR20/50/70 indicators with a medium-quality level evaluation. Tofacitinib treatment at 5 mg for 12 weeks led to significant improvements in ACR20 indicators with a medium-quality evaluation. Secondly, the combination of Baricitinib and Filgotinib with methotrexate treatment also demonstrated considerable efficacy compared to JAK inhibitor monotherapy, evaluated as medium quality using the GEADE scale. However, there is no discernible advantage among various types of JAK inhibitors due to the variability in efficacy and safety across different dosage regimens, rendering it challenging to ascertain drug superiority.

The observed outcomes can be attributed to several factors. Firstly, a scarcity of systematic reviews and research literature pertaining to JAK inhibitors exists, accompanied by notable methodological deficiencies, such as selection bias, publication bias, and others, consequently leading to a low level of evidence in the study. These deficiencies may encompass inadequacies in study design, data collection, and analysis methodologies. Consequently, these shortcomings have the potential to introduce biases such as selection bias and publication bias, thereby diminishing the reliability and validity of the study findings and leading to a diminished level of evidence. Secondly, the variability in pain tolerance levels among subjects introduces subjectivity in the assessment of outcome indicators. This subjectivity contributes to the generation of diverse and heterogeneous results, which in turn compromise the robustness and generalizability of the study outcomes. Finally, the plethora of available JAK inhibitors, coupled with the infrequent overlap in intervention doses selected by included studies, contributes to result heterogeneity. This heterogeneity not only complicates data interpretation but also undermines the comparability and consistency of study outcomes, thereby compromising the overall quality of the investigation.

### 4.4 Safety of JAK inhibitors

In terms of safety, GRADE scale evidence demonstrated that the clinical use of Filgotinib exhibited a lower incidence of adverse reactions such as herpes zoster, upper respiratory tract infection, and nasopharyngitis. Similarly, low-dose Baricitinib also resulted in fewer infections and cases of herpes zoster, indicating a certain level of safety. The selection of 15 mg dose of Upadacitinib showed a reduced occurrence of serious adverse events and infections, suggesting favorable safety profiles. However, several studies have also reported occurrences of infections, herpes zoster, and cardiovascular events subsequent to the administration of JAK inhibitors such as Tofacitinib, thereby diminishing the strength of supporting evidence.

Changes in outcome measures can be ascribed to several factors. Firstly, the utilization of a wide array of JAK inhibitors in clinical practice, developed by different pharmaceutical companies, has led to inconsistent clinical responses. Secondly, the variance in administered doses across the studies included in the analysis has resulted in divergent adverse reactions. Lastly, the incorporation of studies with small sample sizes and wide confidence intervals may introduce publication bias, potentially skewing the overall interpretation of the findings.

### 4.5 Analysis of the contradiction between low score quality and clinical efficacy

The dosage of JAK inhibitor utilized in the study may differ from the clinical dosage, and the pharmaceutical company involved may also vary, leading to significant discrepancies in potential side effects. 2. Individuals of varying ages and physical conditions may exhibit different tolerances to the side effects of JAK inhibitors. Given the wide age range of participants in the study, it is challenging to definitively determine the safety profile of JAK inhibitors. Future research could consider conducting subgroup analyses based on age and health status to more accurately assess the safety profile of JAK inhibitors at specific levels.3. The score results of the outcome indicators included in the study are still satisfactory; however, the low final score can be attributed to methodological defects. In future studies, it is imperative to enhance the rigor of researchers, provide a reasonable and comprehensive explanation for any bias, clearly demonstrate the methods of inclusion and exclusion, and minimize researcher subjectivity.

### 4.6 Limitations

This study also has certain limitations. Firstly, only electronic literature was retrieved, resulting in a reduced number of included literature and potential missed detections, thereby compromising the comprehensiveness of the study. Secondly, although the aforementioned three scales for systematic review/meta-analysis of the evaluation process are relatively detailed and clear, there still exists a subjective judgment by researchers that can influence the final evaluation results. In addition, included non-randomized controlled trials alongside randomized controlled trials may introduce a higher risk of bias, potentially affecting the overall evaluation. Lastly, the quality of the included reviews is generally low and sample sizes are relatively small, which may somewhat undermine the reliability and authenticity of the evaluations.

## 5 Conclusion

In general, the key findings of this study indicate that JAK inhibitors have significant therapeutic effects on RA and are generally safe for clinical practice, while there is room for improvement in the methodological and reporting quality of research on JAK inhibitors for treating RA. Specifically, deficiencies were observed in the formulation and registration of study protocols, lack of justification for inclusion criteria, inadequate comprehensive search strategies leading to insufficient study completeness and consistency, and subjectivity in the criteria for inclusion and exclusion, which decreased the credibility of the research. Therefore, we call for future researchers to conduct deeper analyses in areas and to improve the quality of evidence by reducing the risk of bias in clinical trials.

## Data Availability

The raw data supporting the conclusions of this article will be made available by the authors, without undue reservation.
