# Supplementary material for: Systematic review of Janus kinases inhibitors for rheumatoid arthritis: methodology, reporting, and quality of evidence evaluation
Source: Front Pharmacol. 2024 Sep 25;15:1459511. doi: 10.3389/fphar.2024.1459511 (PMC11461343; doi:10.3389/fphar.2024.1459511)
Supplement: Supplementary file 1 [file Table1.pdf]

Supplementary Table 1 Search strategy for PubMed , Web of Science, Scopus, EMBASE, Livivo

NO.	Title/Abstract
#1	Rheumatoid Arthritis
#2	RA
#3	#1 OR #2
#4	Janus kinases inhibitors
#5	JAK inhibitors
#6	Ruxolitinib, Baricitinib, Tofacitinib, Fedratinib, Momelotinib, Pacritinib, Fligotinib, Upadacitinib, Itacitinib, Decernotinib, Peficitinib, Abrocitinib, Ritlecitinib
#7	#4 OR #5 OR #6
#8	meta-analysis
#9	systematic review
#10	#8 OR #9
#11	#3 AND #6 AND #9

Supplementary Table 2 Search strategy for CNKI, Wanfang and VIP

NO.	Key words (In Chinese)
#1	"类风湿关节炎"
#2	"类风湿性关节炎"
#3	#1 OR #2(Combining the terms for a comprehensive search)
#4	"JAK 抑制剂"
#5	Specific drug names in Chinese phonetics:<br>巴瑞替尼<br>托法替尼<br>卢索替尼<br>非德拉替尼<br>帕西替尼<br>非德替尼<br>菲戈替尼<br>乌帕替尼<br>依他替尼<br>曲赛诺替尼

佩菲替尼	
阿布罗替尼	
利特替尼	
#6	Terms related to systematic review methods:
"元分析"	
"汇总分析"	
"系统评价"	
#7	#5 OR #6
#8	#3 AND #7

Note:VIP, CNKI, and Wan Fang primarily index Chinese-language literature, and searches are conducted using Chinese search terms:类风湿关节炎:(Rheumatoid Arthritis;类风湿性关节炎:Rheumatoid Arthritis;JAK 抑制剂:JAK Inhibitor;巴瑞替尼:Baricitinib;托法替尼:Tofacitinib;卢索替尼:Ruxolitinib);非德拉替尼:Fedratinib;帕西替尼:Pacritinib;非德替尼:Peficitinib;菲戈替尼:Fegitinib;乌帕替尼:Upadacitinib;依他替尼:Itacitinib;曲赛诺替尼:Cerdulatinib;佩菲替尼:Peficitinib;阿布罗替尼:Abrocitinib;利特替尼:Litifatinib.

Supplementary Table 3 Rationale of items of AMSTAR-2

Item	Answer		
1. Did the research questions and inclusion criteria for the review include the components of PICO?	Yes	No	
2.Did the report of the review contain an explicit statement that the review methods were established prior to the conduct of the review and did the report justify any significant deviations from the protocol？	Yes	No	Partial Yes
3. Did the review authors explain their selection of the study designs for inclusion in the review?	Yes	No	
4. Did the review authors use a comprehensive literature search strategy?	Yes	No	Partial Yes
5. Did the review authors perform study selection in duplicate?	Yes	No	
6. Did the review authors perform data extraction in duplicate?	Yes	No	
7. Did the review authors provide a list of excluded studies and justify the exclusions?	Yes	No	Partial Yes
8. Did the review authors describe the included studies in adequate detail?	Yes	No	Partial Yes
9. Did the review authors use a satisfactory technique for assessing the risk of bias [42] in individual studies that were included in the review?	Yes	No	Partial Yes
10. Did the review authors report on the sources of funding for the studies included in the review?	Yes	No	
11. If meta-analysis was performed, did the review authors use appropriate methods for statistical combination of results?	Yes	No	
12. If meta-analysis was performed, did the review authors assess the potential impact of RoB in individual studies on the results of the meta-analysis or other evidence synthesis?	Yes	No	
13. Did the review authors account for RoB in primary studies when interpreting/discussing the results of the review?	Yes	No	
14. Did the review authors provide a satisfactory explanation for, and discussion of, any heterogeneity observed in the results of the review?	Yes	No	
15. If they performed quantitative synthesis did the review authors carry out an adequate investigation of publication bias (small study bias) and discuss its likely impact on the results of the review?	Yes	No	
16. Did the review authors report any potential sources of conflict of interest, including any funding they received for conducting the review?	Yes	No	

Supplementary Table 4 The PRISMA checklists

Section and Topic	ltem	Checklist item
**TITLE**		
Title	1	Identity the repot as a systematic review.
**ABSTRACT**		
Abstract	2	See the PRISMA 2020 for Abstracts checlist.
**INTRODUCTION**		
Rationale	3	Describe the rationale for the review in the context of existing knowledge.
Objectives	4	Provide an explict statement of the objective(s) or question(s) the review addresses.
**METHODS**		
Eligiblity criteria	5	Specify the inclusion and exclusion criteria for the review and how studies were grouped for the syntheses.
information sources	6	Specily all databases, registers, websites, organisations, reference lists and other sources searched or consuted to identity studies. Specily the date when each source was last searched or consulted.
Search strategy	7	Present the full search strategies for all databases, registers and websites, incuding any fiters and limits used.
Selection process	8	Specily the methods used to decide whether a study met the inclusion criteia of the review, including how many reviewers screened each record and each report retrieved, whether they worked independently, and if applicable, details of automatin tools used in the process.
Data collection process	9	Specily the methods used to collct data from reports, including how many reviewers cllclted data from each report, whether they worked independently. any processes for obtaining or confirming data from study investigators, and if applicabie, details of automation tools used in the process.
Data items	10a	List: and define all outcomes for which data were sought Specily whether all results that were compatible with each outcome domain in each study were sought (e.g. for all measures, time points, analyses), and if not, the methods used to decide which result to cllet
	10b	List and detine all other variables for which data were sought (e.g. participant and intervention characterstics, funding sources). Describe any assumptions made about any missing or unclear infomation.
Study risk of bias assessment	11	Specily the methods used to assess risk of bias in the inluded studies, incuding detais of the tol(s) used, how many reviewers assessed each study and whether they worked

		independenly, and if applicable, detaills of automation tools used in the process.
Eeffect measures	12	Specily for each outcome the efict measure(s) (e.g. rik ratio, mean dffrence) used in the synthesis or presentation of rsults
Synthesis methods	13a	Describe the processes used to decide which studies were eigible for each synthesis (eg. tabulating the study intervention characerstics and comparing against the planned groups for each synthesis (item #5)).
	13b	Describe any methods required to prepare the data for presentation or synthesis, such as handing of missing summary satistics, or data conversions.
	13c	Describe any methods used to tabulate or visully display results of individual studies and syntheses.
	13d	Describe any methods used to synthesize result and provide a rationale for the choice(s). f meta analysis was performed, describe the model(s), method(s) to ldentit the presence and extent of statistical heterogeity and software package(s) used.
	13e	Describe any methods used to explore possible causes of heterogeneity among study results (e.g. subgroup analysis, meta regression).
	13f	Describe any sensitvity analyses conducted to assess robustness of the synthesized results.
Reporting bias assessment	14	Describe any methods used to assess risk of bias due to missing result in a synthesis (arising from reporting biases).
Certainty assessment	15	Describe any methods used to assess certainty (or confidence) in the body of evidence for an outcome.
**RESULTS**		
Study selection	16a	Describe the result of the search and selection process, from the number of records identlied in the search to the number of studies incuded in the review, ideall using a flow diagram.
	16b	Cite studies that might appear to meet the inclusion criteria, but which were excluded, and explain why they were excluded.
Study characteristics	17	Cite each included study and present its characteristics.
Risk of bias in studies	18	Present assessments of risk of bias for each included study.
Results of individual studies	19	For all outcomes, present, for each. study: (a) summary statistics for each group (where apropiate) and (b) an efet estimate and its precision (eg. confidence/credibile interval)l, idel using structured tables or plots.
Results of syntheses	20a	For each synthesis, biely summarise the characteristic and risk of bias among contributing studies.

	20b	Present resuls of all statistical syntheses conducted. If meta -analysis was done, present for each the summary estimate and its precision (e.g. confidence/credibie interal) and measures of statistical heterogeneity. If comparing groups, describe the direcion of the efect.
	20c	Present results of all investigations of possible causes of heterogeneity among study results.
	20d	Present results of all sensitivit analyses conducted to assess the robustness of the synthesized results.
Reporting biases	21	Present assessments of risk of bias due to missing results (arising from reporting biases) for each synthesis assessed.
Certainty of evidence	22	Present assessments of certainty (or confidence) in the body of evidence for each outcome assessed.
**DISCUSSION**		
Discussion	23a	Provide a general interpretation of the results in the contxt of other evidence.
	23b	Discuss any litations of the evidence included in the review.
	23c	Discuss any limitations of the review processes used.
	23d	Discuss implictions of the results for practice, policy, and future research.
**OTHER INFORMATION**		
Registration and protocol	24a	Provide registration information for the review, including register name and registration number, or state that the review was not registered.
	24b	Indicate where the review protocol can be accessed, or state that a protocol was not prepared.
	24c	Describe and explain any amendments to information provided at registration or in the protocol.
Support	25	Describe sources of financlal or non-inancial support for the review, and the role of the funders or sponsors in the review.
Competing interests	26	Declare any competing interests of review authors.
Avaiabilty of data, code and other materials	27	Report which of the fllwing are publicy available and where they can be found: template data collection forms; data extracted frm included studies; data used for all analyses: analytic code; any other materials used in the review.

Supplementary Table 5 Rating of GRADE system

Study design	Quality of evidence	Specific explanation	Lower if	Total sore
Randomized trial	high	Further research is very unlucky to our confidence in the estimate of effect	Risk of bias<br>-1 serious<br>-2 very serious	≥0
	Medium	Further research is likely to have an important impact on our confidence in the estimate of effect and may change the estimate	Inconsistency<br>-1 serious<br>-2 very serious<br>Indirectness<br>-1 serious	-1
Observational study	low	Further research is very likely to have an important impact on our confidence in the estimate of effect and may change the estimate	-2 very serious<br>Imprecision<br>-1 serious<br>-2 very serious<br>Publication	-2
	very low	Any estimate of effect is very uncertain	bias<br>-1 serious<br>-2 very serious	≤-3

Supplementary Table 6 The evaluation results of the included reviews of GRADE system

Study	Years	RCT/ Patients	Outcomes	Effect index	95%CI	I^2	Risk of bias	inconsistency	indirectness	imprecision	Publication bias	Quality
**Zhao**	2022	5/3544	**ACR20**									
			Filgotinib 50mg	RR=1.68	0.94,3.00	82	-1	-1	0	-1	0	Critically low
			Filgotinib 100mg	RR=1.50	1.26,1.78	75	-1	-1	0	0	0	Low
			Filgotinib 200mg	RR=1.66	1.37,2.01	83	-1	-1	0	0	0	Low
			**ACR50**									
			Filgotinib 50mg	RR=2.51	1.59,3.97	0	-1	0	0	0	0	Medium
			Filgotinib 100mg	RR=1.91	1.57,2.34	38	-1	0	0	0	0	Medium
			Filgotinib 200mg	RR=2.41	1.93,3.00	54	-1	-1	0	0	0	Low
			**ACR70**									
			Filgotinib 50mg	RR=2.19	1.02,4.66	0	-1	0	0	0	0	Medium
			Filgotinib 100mg	RR=2.46	1.96,3.09	0	-1	0	0	0	0	Medium
			Filgotinib 200mg	RR=3.05	1.93,3.00	0	-1	0	0	0	0	Medium
			**DAS28-CRP≤3.2**									
			Filgotinib 50mg	RR=1.68	0.82,3.44	0	-1	0	0	-1	0	Low
			Filgotinib 100mg	RR=1.77	1.55,2.03	0	-1	0	0	0	0	Medium
			Filgotinib 200mg	RR=2.12	1.88,2.40	0	-1	0	0	0	0	Medium
			**DAS28-CRP＜2.6**									
			Filgotinib 50mg	RR=1.77	0.87,3.60	0	-1	0	0	-1	-1	Critically low
			Filgotinib 100mg	RR=2.35	1.93,2.86	3	-1	0	0	0	-1	Low
			Filgotinib 200mg	RR=2.84	2.38,3.38	27	-1	0	0	0	0	Medium
			**Adverse Events**									
			SAE	RR=0.98	0.76, 1.26	0	-1	0	0	-1	0	Low
			Infection	RR=1.13	1.03,1.24	40	-1	0	0	-1	0	Low

Serious Infection	RR=1.17	0.72,1.90	0	-1	0	0	0	0	Medium
herpes zoster	RR=1.37	0.71,2.66	0	-1	0	0			Medium
upper respiratory infection	RR=1.50	1.11,2.02	19	-1	0	0	0	0	Medium
nasopharyngitis	RR=1.29	0.99,1.68	0	-1	0	0			Medium
hemoglobin decreased									
Filgotinib 50mg	RR=0.92	0.61,1.39	Not Mentioned	-1	-1	0	-1	0	Critically low
Filgotinib 100mg	RR=0.50	0.36,0.69	Not Mentioned	-1	-1	0	0	0	Low
Filgotinib 200mg	RR=0.60	0.44,0.82	Not Mentioned	-1	-1	0	-1	0	Critically low
neutropenia									
Filgotinib 50mg	RR=0.25	0.04，1.49	Not Mentioned	-1	-1	0	-1	0	Critically low
Filgotinib 100mg	RR=1.42	0.77，2.62	Not Mentioned	-1	-1	0	-1	0	Critically low
Filgotinib 200mg	RR=2.12	1.18，3.79	Not Mentioned	-1	-1	0	0	0	Low
lymphocytopenia									
Filgotinib 50mg	RR=1.10	0.51,2.39	Not Mentioned	-1	-1	0	-1	0	Critically low
Filgotinib 100mg	RR=0.79	0.50,1.26	Not Mentioned	-1	-1	0	-1	0	Critically low
Filgotinib 200mg	RR=1.39	0.94,2.06	Not Mentioned	-1	-1	0	-1	0	Critically low
thrombocytopenia									

			Filgotinib 50mg	RR=0.30	0.01,7.14	Not Mentioned	-1	-1	0	-1	0	Critically low
			Filgotinib 100mg	RR=1.18	0.42,3.28	Not Mentioned	-1	-1	0	-1	0	Critically low
			Filgotinib 200mg	RR=0.88	0.29,2.68	Not Mentioned	-1	-1	0	-1	0	Critically low
**Zhang**	2018	9/3742	**ACR20**									
			Tofacitinib 5mg vs placebo									
			3 month	OR=3.77	2.74,5.18	59	-1	-1	0	0	-1	Critically low
			6 month	OR=2.48	1.87,3.29	33	-1	0	0	0	-1	Low
			Tofacitinib 10mg vsplacebo									
			3 month	OR=4.52	3.75,5.44	48	-1	0	0	0	-1	Low
			6 month	OR=3.34	2.67,4.18	27	-1	0	0	0	-1	Low
			Tofacitinib 5mg vs 10mg									
			Tofacitinib 3month	OR=1.27	1.10,1.48	Not Mentioned	-1	-1	0	0	-1	Critically low
			Tofacitinib 6month	OR=1.26	1.07,1.49	Not Mentioned	-1	-1	0	0	-1	Critically low
			**ACR50**									
			Tofacitinib 5mg vs placebo									
			3 month	OR=3.89	3.06,4.95	Not Mentioned	-1	-1	0	0	0	Low
			6 month	OR=3.44	2.59,4.56	Not Mentioned	-1	-1	0	0	0	Low
			Tofacitinib 10mg vs placebo								0	
			3 month	OR=4.90	3.81,6.32	Not	-1	-1	0	0	0	Low

			Mentioned						
6 month	OR=4.16	3.15,5.50	Not Mentioned	-1	-1	0	0	0	Low
Tofacitinib 5mg vs 10mg								0	
3 month	OR=1.16	0.99,1.36	Not Mentioned	-1	-1	0	-1	0	Critically low
6 month	OR=1.15	0.97,1.37	Not Mentioned	-1	-1	0	-1	0	Critically low
**ACR70**								0	
Tofacitinib 5mg vs placebo								0	
3 month	OR=4.87	3.18,7.46	Not Mentioned	-1	-1	0	0	0	Low
6 month	OR=5.47	3.35,8.95	Not Mentioned	-1	-1	0	0	0	Low
Tofacitinib 10mg vsplacebo								0	
3 month	OR=6.62	4.36,10.06	Not Mentioned	-1	-1	0	0	0	Low
6 month	OR=7.58	4.68,12.27	Not Mentioned	-1	-1	0	0	0	Low
Tofacitinib 5mg vs 10mg								0	
3 month	OR=1.41	1.14,1.74	Not Mentioned	-1	-1	0	0	0	Low
6 month	OR=1.35	1.08,1.68	Not Mentioned	-1	-1	0	0	0	Low
SAE	OR=0.94	0.58,1.51	Not Mentioned	-1	-1	0	-1	0	Critically low

			AE	OR=1.43	1.02,2.01	Not Mentioned	-1	-1	0	0	0	Low
			nausea	OR=1.18	0.16,2.09	Not Mentioned	-1	-1	0	-1	0	Critically low
			diarrhea	OR=1. 90	1.15,3.12	Not Mentioned	-1	-1	0	0	0	Low
			nasopharyngitis	OR=1.25	0.80,1.94	Not Mentioned	-1	-1	0	-1	0	Critically low
			upper respiratory infection	OR=1.41	0.89,2.24	Not Mentioned	-1	-1	0	-1	0	Critically low
			urinary tract infection	OR=1.88	0.99,3.59	Not Mentioned	-1	-1	0	-1	0	Critically low
			bronchitis	OR=1.24	0.49,3.09	Not Mentioned	-1	-1	0	-1	0	Critically low
			herpes zoster	OR=1.08	0.15,7.81	Not Mentioned	-1	-1	0	-1	0	Critically low
			arthrodynia	OR=0.48	0.22,1.06	Not Mentioned	-1	-1	0	-1	0	Critically low
**Qin**	2018	6/3546	**ACR20**									
			Baricitinib 2mg vs placebo	OR=2.81	2.10,3.76	0	-1	0	0	0	-1	Low
			Baricitinib 4mg vs placebo	OR=3.16	2.68,3.74	37	-1	0	0	0	-1	Low
			Baricitinib 4mg vs 2mg	OR=0.98	0.65,1.49	53	-1	-1	0	-1	-1	Critically low
			**ACR50**									
			Baricitinib 2mg vs placebo	OR=3.23	2.20,4.76	27	-1	0	0	0	0	Medium
			Baricitinib 4mg vs placebo	OR=3.34	2.45,4.55	43	-1	0	0	0	0	Medium

			Baricitinib 4mg vs 2mg	OR=1.20	0.73,1.95	30	-1	0	0	-1	0	Low
			**ACR70**									
			Baricitinib 2mg vs placebo	OR=8.16	4.00,16.68	15	-1	0	0	0	0	Medium
			Baricitinib 4mg vs placebo	OR=4.42	2.50,7.82	21	-1	0	0	0	0	Medium
			Baricitinib 4mg vs 2mg	OR=0.93	0.63,1.36	14	-1	0	0	-1	0	Low
			**AE**	OR=1.34	1.14,159	31	-1	0	0	0	0	Medium
			**SAE**	OR=1.00	0.65,1.53	0	-1	0	0	-1	0	Low
			Withdrawal due to AE	OR=1.44	0.92,2.24	8	-1	0	0	-1	0	Low
			**Serious Infection**	OR=1.30	0.46,3.68	13	-1	0	0	-1	0	Low
			**Infection**	OR=1.46	1.19,1.79	0	-1	0	0	0	0	Medium
			herpes zoster	OR=3.91	1.38,11.07	0	-1	0	0	0	0	Medium
**Liu**	2020	5/1773	**ACR20**									
			Peficitinib 25mg vs placebo	RR=1.07	0.66,1.73	Not Mentioned	-1	-1	0	-1	-1	Critically low
			Peficitinib 50mg vs placebo	RR=1.51	1.04,2.20	Not Mentioned	-1	-1	0	0	-1	Critically low
			Peficitinib 100mg vs placebo	RR=2.01	1.29,3.13	Not Mentioned	-1	-1	0	0	-1	Critically low
			Peficitinib 150mg vs placebo	RR=2.39	1.58,3.61	Not Mentioned	-1	-1	0	0	-1	Critically low
			**ACR50**									
			Peficitinib 25mg vs placebo	RR=0.02	0.55,1.56	Not Mentioned	-1	-1	0	-1	-1	Critically low
			Peficitinib 50mg vs placebo	RR=1.48	0.97,2.25	Not Mentioned	-1	-1	0	-1	-1	Critically low
			Peficitinib 100mg vs placebo	RR=2.87	1.61,5.11	Not	-1	-1	0	0	-1	Critically low

			Mentioned						
Peficitinib 150mg vs placebo	RR=3.52	1.78,6.96	Not Mentioned	-1	-1	0	0	-1	Critically low
**ACR70**									
Peficitinib 25mg vs placebo	RR=0.79	0.36,1.71	Not Mentioned	-1	-1	0	-1	-1	Critically low
Peficitinib 50mg vs placebo	RR=1.54	0.80,2.94	Not Mentioned	-1	-1	0	-1	-1	Critically low
Peficitinib 100mg vs placebo	RR=3.50	1.62,7.58	Not Mentioned	-1	-1	0	0	-1	Critically low
Peficitinib 150mg vs placebo	RR=4.59	1.47,14.30	Not Mentioned	-1	-1	0	0	-1	Critically low
**DAS28-ESR＜2.6**	RR=4.83	3.20,7.28	13.5	-1	0	0	0	-1	Low
Peficitinib 25mg vs placebo	RR=1.16	0.31,4.25	Not Mentioned	-1	-1	0	-1	-1	Critically low
Peficitinib 50mg vs placebo	RR=1.41	0.41,4.87	Not Mentioned	-1	-1	0	-1	-1	Critically low
Peficitinib 100mg vs placebo	RR=5.37	2.68,10.77	Not Mentioned	-1	-1	0	0	-1	Critically low
Peficitinib 150mg vs placebo	RR=7.44	3.78,14.65	Not Mentioned	-1	-1	0	0	-1	Critically low
**DAS28-CRP＜2.6**	RR=3.41	2.65,4.39	49.1	-1	0	0	-1	-1	Critically low
Peficitinib 25mg vs placebo	RR=0.48	0.16,1.48	Not Mentioned	-1	-1	0	-1	-1	Critically low
Peficitinib 50mg vs placebo	RR=1.29	0.54,7.07	Not Mentioned	-1	-1	0	-1	-1	Critically low

			Peficitinib 100mg vs placebo	RR=4.00	2.67,5.99	Not Mentioned	-1	-1	0	0	-1	Critically low
			Peficitinib 150mg vs placebo	RR=4.45	2.99,6.63	Not Mentioned	-1	-1	0	0	-1	Critically low
			SAE	RR=0.90	0.44,1.86	0	-1	0	0	-1	0	Low
			**diarrhoea**	RR=1.12	0.47,2.67	0	-1	0	0	-1	0	Low
			upper respiratory infection	RR=0.94	0.39,2.24	0	-1	0	0	-1	0	Low
			**Urinary tract infections**	RR=0.82	0.36,1.84	0	-1	0	0	-1	0	Low
			**dyspepsia**	RR=1.44	0.26,7.94	0	-1	0	0	-1	0	Low
			headache	RR=1.70	0.39,7.32	0	-1	0	0	-1	0	Low
			nasopharyngitis	RR=1.87	0.75,4.62	1.1	-1	0	0	-1	0	Low
**Yao**	2020	7/4208	**ACR20**	RR=1.69	1.58,1.81	24	-1	0	0	0	0	Medium
			**ACR50**	RR=2.53	2.03,3.15	58	-1	-1	0	0	0	Low
			**ACR70**	RR=4.20	2.91,6.07	46	-1	0	0	0	0	Medium
			**DAS28≤3.2**	RR=2.41	2.17,2.67	30	-1	0	0	0	0	Medium
			**DAS28＜2.6**	RR=3.02	2.60,3.51	34	-1	0	0	0	0	Medium
			**CDAI≤10**	RR=2.06	1.69,2.51	58	-1	-1	0	0	0	Low
			**CDAI≤2.8**	RR=3.16	2.52,3.97	49	-1	0	0	0	0	Medium
			**SDAI≤11**	RR=3.47	2.23,5.39	19	-1	0	0	0	0	Medium
			**AE**	RR=1.15	1.09,1.22	19	-1	0	0	0	0	Medium
			**SAE**	RR=1.62	1.11,2.38	8	-1	0	0	0	0	Medium
			**Infection**	RR=1.26	1.13,1.40	48	-1	0	0	0	0	Medium
			**Serious Infection**	RR=1.60	0.85,3.01	0	-1	0	0	-1	0	Low
			herpes zoster	RR=1.73	0.86,3.48	0	-1	0	0	-1	0	Low
			**Liver disease**	RR=1.29	0.92,1.81	0	-1	0	0	-1	0	Low

		**CA**	RR=1.00	0.34,2.94	0	-1	0	0	-1	0	Low
		**Cardiovascular events**	RR=1.42	0.37,5.48	0	-1	0	0	-1	0	Low
		**Death**	RR=0.53	0.11,2.58	0	-1	0	0	-1	0	Low
**Liu**	8/2909	**HAQ-DI**									
		5mg	WMD=-0.29	-0.34,-0.24	Not Mentioned	-1	-1	0	0	-1	Critically low
		10mg	WMD=-0.35	-0.40,-0.30	Not Mentioned	-1	-1	0	0	-1	Critically low
		**ACR20(3 month)**									
		5mg	RR=1.84	1.18,2.86	Not Mentioned	-1	-1	0	0	-1	Critically low
		10mg	RR=2.04	1.29,3.23	Not Mentioned	-1	-1	0	0	-1	Critically low
		**ACR50(3 month)**									
		5mg	RR=2.93	2.27,3.78	Not Mentioned	-1	-1	0	0	-1	Critically low
		10mg	RR=3.20	2.49,4.13	Not Mentioned	-1	-1	0	0	-1	Critically low
		**ACR70(3 month)**									
		5mg	RR=2.93	2.27,3.78	Not Mentioned	-1	-1	0	0	-1	Critically low
		10mg	RR=4.40	2.89,6.71	Not Mentioned	-1	-1	0	0	-1	Critically low
		**DAS28-4(ESR)＜2.6 (3 month)**									
		5mg	RR=2.93	2.27,3.78	Not	-1	-1	0	0	-1	Critically low

			Mentioned						
10mg	RR=3.09	1.55,6.17	Not Mentioned	-1	-1	0	0	-1	Critically low
**ACR20(6 month)**									
5mg	RR=1.83	1.53,2.19	Not Mentioned	-1	-1	0	0	-1	Critically low
10mg	RR=2.11	1.77,2.51	Not Mentioned	-1	-1	0	0	-1	Critically low
**ACR50(6 month)**									
5mg	RR=2.12	1.74,2.57	Not Mentioned	-1	-1	0	0	-1	Critically low
10mg	RR=2.01	1.66,2.44	Not Mentioned	-1	-1	0	0	-1	Critically low
**ACR70(6 month)**									
5mg	RR=4.12	1.69,10.06	Not Mentioned	-1	-1	0	0	-1	Critically low
10mg	RR=5.70	1.81,17.97	Not Mentioned	-1	-1	0	0	-1	Critically low
**DAS28-4(ESR)＜2.6 (6 month)**									
5mg	RR=4.89	1.97,12.15	Not Mentioned	-1	-1	0	0	-1	Critically low
10mg	RR=9.85	4.05,23.97	Not Mentioned	-1	-1	0	0	-1	Critically low
AE	RR=1.00	0.94,1.07	Not Mentioned	-1	-1	0	-1	-1	Critically low
SAE	RR=0.62	0.31,1.24	Not	-1	-1	0	-1	-1	Critically low

						Mentioned					
**Li**	2019	8/2738	**ACR20**								
			Upadacitinib	OR＝3.38	2.71,4.22	Not Mentioned	-1	-1	0	0	-1	Critically low
			Filgotinib	OR＝3.34	2.40,4.66	Not Mentioned	-1	-1	0	0	-1	Critically low
			**ACR50**	OR=3.78	2.98,4.78	0	-1	0	0	0	0	Medium
			Upadacitinib	OR＝3.68	2.78,4.86	Not Mentioned	-1	-1	0	0	0	Low
			Filgotinib	OR＝4.03	2.58,6.29	Not Mentioned	-1	-1	0	0	0	Low
			**ACR70**	OR=4.31	3.05,6.09	0	-1	0	0	0	0	Medium
			**DAS28＜3.2**	OR=3.86	2.98,5.00	37	-1	0	0	0	0	Medium
			**AE**	OR=1.33	1.11,1.61	6	-1	0	0	0	-1	Low
			**SAE**	OR=1.08	0.65,1.80	22	-1	0	0	-1	0	Low
			**Infection**	OR=1.43	1.12,1.81	0	-1	0	0	0	0	Medium
			**Serious Infection**	OR=1.23	0.46,3.25	0	-1	0	0	-1	0	Low
			herpes zoster	OR=1.02	0.39,2.66	0	-1	0	0	-1	0	Low
			**Liver disease**	OR=1.46	0.64,3.31	0	-1	0	0	-1	0	Low
**Kunwar**	2018	5/2458	**baricitinib 2mg vs placebo**								
			ACR20	OR=2.09	1.60,2.71	0	-1	-1	0	0	-1	Critically low
			ACR50	OR=2.30	1.68,3.15	0	-1	-1	0	0	-1	Critically low
			ACR70	OR=4.05	2.54,6.44	0	-1	-1	0	0	0	Low
			DAS28-CRP＜2.6	OR=3.20	2.14,4.78	0	-1	-1	0	0	0	Low

			SDAI≤3.3	OR=4.56	2.43,8.54	0	-1	-1	0	0	0	Low
			**baricitinib 4mg vs 2mg**									
			ACR20	OR=1.29	0.88,1.89	43	-1	0	0	-1	-1	Critically low
			ACR50	OR=1.34	0.94,1.92	28	-1	0	0	-1	-1	Critically low
			ACR70	OR=1.34	0.76,2.38	56	-1	0	0	-1	0	Low
			DAS28-CRP＜2.6	OR=1.83	0.95,3.52	71	-1	0	0	-1	0	Low
			SDAI≤3.3	OR=1.46	0.68,3.13	59	-1	0	0	-1	0	Low
			**baricitinib 2mg vs placebo**									
			Any adverse events	OR=1.03	0.80,1.34	0	-1	0	0	-1	0	Low
			Serious adverse events	OR=0.68	0.37,1.27	0	-1	0	0	-1	0	Low
			Serious infections	OR=0.98	0.27,3.49	31	-1	0	0	-1	0	Low
			Herps Zoster	OR=2.34	0.27,20.47	37	-1	0	0	-1	0	Low
**Lilla**	2022	33/24135	AC R50 mesponse	OR=3.79	3.22,4.46	56.6	-1	0	0	0	0	Medium
			AC R70 response	OR=4.05	3.28,5.01	71.2	-1	0	0	0	0	Medium
			HAQ-DI improvement	OR=2.19	1.81,2.64	43.3	-1	0	0	0	0	Medium
			CDAI remission	OR=3.41	2.67,4.36	57	-1	0	0	0	0	Medium
			SDAI remission	OR=3.58	2.76,4.75	11.5	-1	0	0	0	0	Medium
			DAS28CRP remission	OR=3.75	3.08,4.56	48.7	-1	0	0	0	0	Medium
			DAS28-ESR remission	OR=3.99	2.84,5.59	18.6	-1	0	0	0	0	Medium
			Side effects	OR=1.20	1.07,1.34	36.5	-1	0	0	0	0	Medium
			Serbous side effects	OR=0.95	0.75,1.21	0	-1	0	0	-1	0	Low
			Discon thuation	OR=1.03	0.81,1.31	0	-1	0	0	-1	0	Low
			Deaths	OR=0.81	0.40,1.63	0	-1	0	0	0	0	Medium
			Swollen joint count	WMD=-3.54	-4.17,-2.90	57	-1	0	0	0	0	Medium

Tender joint count	WMD=-5.52	-6.16,-4.88	25.3	-1	0	0	0	0	Medium
CRP	WMD=-8.57	-10.14,-6.99	97.1	-1	0	0	0	0	Medium
ESR	WMD=-14.17	-19.28 ,-9.07	90.3	-1	0	0	0	0	Medium
HAQ–DI	WMD=-0.3	-0.34 ,-0.26	70.8	-1	0	0	0	0	Medium
DAS28–CRP	WMD=-1.2	-1.36,-1.05	84.0	-1	0	0	0	0	Medium
DAS28–ESR	WMD=-1.22	-1.42,-1.02	65.9	-1	0	0	0	0	Medium
CDAI	WMD=-9.24	-11.18,-7.31	59.2	-1	0	0	0	0	Medium
SDAI	WMD=-9.73	-10.94,-8.52	0	-1	0	0	0	0	Medium
MOS–Sleep	WMD=-3.89	-5.18,-2.60	0	-1	0	0	0	0	Medium
WPAIAI	WMD=-7.32	-10.29,-4.35	0	-1	0	0	0	0	Medium
WPAIA	WMD=-4.98	-7.89,-2.07	0	-1	0	0	0	0	Medium
WPAIOWI	WMD=-9.95	-13.48,6.42	0	-1	0	0	0	0	Medium
WPAIP	WMD=-8.55	-11.75,-5.36	0	-1	0	0	0	0	Medium
MJSduration	WMD=-40.	-59.43,-22.	82.7	-1	0	0	0	0	Medium

				98	53							
			SF–36MCS	WMD=1.99	1.04,2.93	54.2	-1	0	0	0	0	Medium
			SF–36PCS	WMD=4.12	3.67,4.57	0	-1	0	0	0	0	Medium
			FACIT–F	WMD=4.18	3.44,4.92	57.4	-1	0	0	0	0	Medium
			EQ–5D(US)	WMD=0.06	0.04 ,0.07	2.4	-1	0	0	0	0	Medium
			EQ–5D(UK)	WMD=8	0.07 ,0.10	0	-1	0	0	0	0	Medium
			EQ–5D(VAS)	WMD=7.91	6.35 ,9.47	0	-1	0	0	0	0	Medium
**Liu**	2022	3/2290	**52 weeks**									
			ACR20	RD=0.032	-0.027 ,0.091	44.7	-1	-1	0	0	0	Low
			ACR50	RD=0.050	0.003 ,0.097	0.0	-1	0	0	0	0	Medium
			ACR 70	RD=0.056	0.012 ,0.100	0.0	-1	0	0	0	0	Medium
			SDAI≤11	RD=0.043	-0.015 ,0.101	0.0	-1	0	0	0	0	Medium
			CDAI< 10	RD=0.042	-0.016 ,0.100	0.0	-1	0	0	0	0	Medium
			DAS28-4 (ESR)≤3.2	RD=0.055	0.002 ,0.107	0.0	-1	0	0	0	0	Medium
			DAS28-4(CRP)≤3.2	RD=0.049	0.003 ,0.095	0.0	-1	0	0	0	0	Medium
			SDAI≤3.3	RD=0.050	0.008 ,0.092	13.9	-1	0	0	0	0	Medium
			CDAI≤2.8	RD=0.054	0.015 ,0.0	0.0	-1	0	0	0	0	Medium

		93							
DAS28-4 (ESR)<2.6	RD=0.043	0.001 ,0.085	0.0	-1	0	0	0	0	Medium
DAS28-4(CRP)<2.6	RD=0.063	0.018 ,0.108	0.0	-1	0	0	0	0	Medium
HAQ-DI improvement > 0.22	RD=0.001	-0.024 ,0.026	0.0	-1	0	0	0	0	Medium
**24 weeks**									
ACR20	RD=0.047	0.006 ,0.0887	0.0	-1	-1	0	0	0	Low
ACR 50	RD=0.054	0.007 ,0.101	0.0	-1	0	0	0	0	Medium
ACR70	RD=0.039	-0.010 ,0.089	20.2	-1	0	0	0	0	Medium
SDAI <11	RD=0.04	-0.021 ,0.100	7.4	-1	0	0	0	0	Medium
CDAI< 10	RD=0.037	-0.028 ,0.102	17.3	-1	0	0	0	0	Medium
DAS28-4 (ESR)≤3.2	RD=0.051	-0.000 ,0.103	0.0	-1	0	0	0	0	Medium
DAS28-4(CRP)≤3.2	RD=0.058	0.011 ,0.104	0.0	-1	0	0	0	0	Medium
SDAI≤3.3	RD=0.035	0.000 ,0.070	0.0	-1	0	0	0	0	Medium
CDAI<2.8	RD=0.035	-0.000 ,0.070	0.0	-1	0	0	0	0	Medium

DAS28-4 (ESR)<2.6	RD=0.019	-0.021 ,0.059	0.0	-1	0	0	0	0	Medium
DAS28-4 (CRP)<2.6	RD=0.079	0.020 ,0.138	39.1	-1	0	0	0	0	Medium
HAQ-DI improvement > 0.22	RD=0.00	-0.025 ,0.024	0.0	-1	0	0	0	0	Medium
**Safety outcomes (52 weeks)**									
TEAEs	RD=0.056	0.014 ,0.099	0	-1	0	0	0	0	Medium
SAEs	RD=0.014	-0.040 ,0.012	0	-1	-1	0	0	0	Low
AEs leading to study discontinuation	RD=0.032	0.007 ,0.057	22	-1	0	0	0	0	Medium
Deaths	RD=0.000	-0.007 ,0.007	33.4	-1	0	0	0	0	Medium
Serious infections	RD=0.000	-0.022 ,0.023	52.1	-1	0	0	0	0	Medium
Herpes zoster	RD=0.004	-0.009 ,0.016	0	-1	0	0	0	0	Medium
Opportunistic infections	RD=0.000	-0.006 ,0.006	0	-1	0	0	0	0	Medium
Malignancy	RD=0.002	-0.010 ,0.014	61.1	-1	0	0	0	0	Medium
VTE	RD=0.000	-0.005 ,0.005	0	-1	0	0	0	0	Medium
MACE	RD=0.001	-0.005 ,0.0	0	-1	0	0	0	0	Medium

					04							
**Yin**	2021	28/14500	**ACR20**	OR=3.79	3.14,4.59	73	-1	0	0	0	0	Medium
			Baricinib	OR=3.02	2.54,3.60	0	-1	0	0	0	0	Medium
			Decernotinib	OR=4.77	2.42,9.39	48	-1	0	0	0	0	Medium
			Filgotinib	OR=3.39	2.18,5.29	60	-1	-1	0	0	0	Low
			Peficitinib	OR=3.65	1.29,10.29	90	-1	-1	0	0	0	Low
			Tofacitinib	OR=5.67	3.25,9.88	85	-1	-1	0	0	0	Low
			Upadacitinib	OR=3.35	2.78,4.04	0	-1	0	0	0	0	Medium
			**ACR50**	OR=3.83	3.29,4.46	24	-1	0	0	0	0	Medium
			Baricinib	OR=3.77	3.03,4.68	0	-1	0	0	0	0	Medium
			Decernotinib	OR=6.63	3.15,13.97	0	-1	0	0	0	0	Medium
			Filgotinib	OR=3.72	2.62,5.29	0	-1	0	0	0	0	Medium
			Peficitinib	OR=3.32	0.93,11.89	89	-1	-1	0	-1	0	Critically low
			Tofacitinib	OR=3.96	2.68,5.86	0	-1	0	0	0	0	Medium
			Upadacitinib	OR=4.06	3.22,5.12	0	-1	0	0	0	0	Medium
			**ACR70**	OR=5.46	3.78,7.89	0	-1	0	0	0	0	Medium
			Baricinib	OR=5.77	1.78,18.74	0	-1	0	0	0	0	Medium
			Decernotinib	OR=3.21	1.95,5.29	0	-1	0	0	0	0	Medium
			Filgotinib	OR=3.69	0.93,14.59	0	-1	0	0	-1	0	Low
			Peficitinib	OR=4.62	2.46,8.66	76	-1	-1	0	0	0	Low
			Tofacitinib	OR=5.42	2.92,10.07	0	-1	0	0	0	0	Medium
			Upadacitinib	OR=4.63	3.61,5.95	70	-1	-1	0	0	0	Low
**Sung**	2021	19/3442	ACR20	Not Mentioned	0.46,0.65	Not Mentioned	-1	-1	0	-1	0	Critically low

			ACR50	Not Mentioned	0.19,0.29	Not Mentioned	-1	-1	0	-1	0	Critically low
			AE	Not Mentioned	0.53,0.69	Not Mentioned	-1	-1	0	-1	0	Critically low
			SAE	Not Mentioned	0.03,0.07	Not Mentioned	-1	-1	0	-1	0	Critically low
			Withdrawal due to AE	Not Mentioned	0.03,0.06	Not Mentioned	-1	-1	0	-1	0	Critically low
**Sung**	2021	5/1422	ACR20	OR=4.19	2.49,7.06	Not Mentioned	-1	-1	0	0	-1	Critically low
			AE	RD=0.001	-0.05,0.05	Not Mentioned	-1	-1	0	0	-1	Critically low
			SAE	RD=0.005	-0.01,0.02	Not Mentioned	-1	-1	0	0	-1	Critically low
**Zhang**	2013	10/4929	**ACR20 (12 weeks)**									
			Tofacitinib 5mg vs backgroung therapy	RR=2.07	1.80,2.38	51	-1	0	0	0	0	Medium
			Tofacitinib 10mg vs backgroung therapy	RR=2.26	1.97,2.60	38	-1	0	0	0	0	Medium
			Tofacitinib 5mg vs placebo therapy	RR=2.65	2.06,3.39	51	-1	0	0	0	0	Medium
			Tofacitinib 10mg vs placebo therapy	RR=3.00	2.35,3.83	60	-1	-1	0	0	0	Low
			**SAE**									
			Tofacitinib 5mg vs backgroung	RR=1.21	0.71,2.05	10	-1	0	0	-1	-1	Critically low

			therapy									
			Tofacitinib 10mg vs backgroung therapy	RR=1.02	0.59,1.77	11	-1	0	0	-1	-1	Critically low
			Tofacitinib 5mg vs placebo therapy	RR=0.26	0.08,0.79	52	-1	0	0	-1	-1	Critically low
			Tofacitinib 10mg vs placebo therapy	RR=0.52	0.21,1.29	0	-1	0	0	-1	-1	Critically low
**Wang**	2020	20/8982	**ACR20**									+++-
			Tofacitinib 5 mg bid	RR=2.16	1.81,2.58	52.2	-1	-1	0	0	0	Low
			Tofacitinib I0 mg bid	RR=2.48	1.97,3.14	63.2	-1	-1	0	0	0	Low
			Baricitinib 2 mg qd	RR=1.73	1.38,2.16	55.4	-1	-1	0	0	0	Low
			Baricitinib 4 mg qd	RR=1.85	1.63,2.1	36.3	-1	-1	0	0	0	Low
			Upadacitinib 15 mg qd	RR=1.96	1.68,2.28	2.4	-1	-1	0	0	0	Low
			Upadacitinib 30 mg qd	RR=1.90	1.61,2.23	0	-1	-1	0	0	0	Low
			**HAQ-DI**									
			Tofacitinib 5 mg bid	RR=-0.30	-0.361,-0.24	0	-1	0	0	0	0	Medium
			Tofacitinib I0 mg bid	RR=-0.38	-0.44,-0.31	0	-1	0	0	0	0	Medium
			Baricitinib 2 mg qd	RR=-0.19	-0.30,-0.07	8.1	-1	0	0	0	0	Medium
			Baricitinib 4 mg qd	RR=-0.26	-0.37,-0.15	0	-1	0	0	0	0	Medium
			Upadacitinib 15 mg qd	RR=-0.32	-0.37,-0.26	0	-1	0	0	0	0	Medium
			Upadacitinib 30 mg qd	RR=-0.29	-0.37,-0.20	0	-1	0	0	0	0	Medium
			**Adverse events**									
			Tofacitinib 5 mg bid	RR=1.05	0.97,1.13	0	-1	0	0	-1	0	Low
			Tofaitinib I0 mg bid	RR=1.07	0.99,1.15	1.3	-1	0	0	-1	0	Low

	Baricitinib 2 mg qd	RR=1.01	0.92,1.01	0	-1	0	0	-1	0	Low
	Baricitinib 4 mg qd	RR=1.13	1.02,1.24	46.4	-1	0	0	0	0	Medium
	Upadacitinib 15 mg qd	RR=1.14	1.02,1.27	41.4	-1	0	0	0	0	Medium
	Upadacitinib 30 mg qd	RR=1.15	1.02,1.30	0	-1	0	0	0	0	Medium
	**Serious adverse events**									
	Tofaitinib 5 mg bid	RR=0.95	0.44,2.05	4.24	-1	0	0	-1	0	Low
	Tofacitinib 10 mg bid	RR=1.06	0.66,1.70	0	-1	0	0	-1	0	Low
	Baricitinib 2 mg qd	RR=0.69	0.38,1.25	0	-1	0	0	-1	0	Low
	Baricitinib 4 mg qd	RR=1.12	0.77,1.63	0	-1	0	0	-1	0	Low
	Upadacitinib 15 mg qd	RR=1.79	0.74,4.31	43.6	-1	0	0	-1	0	Low
	Upadacitinib 30 mg qd	RR=4.48	0.15,129.9<br>8	79.9	-1	-1	0	-2	0	Critically low
	**Infections**									
	Tofacitinib 5 mg bid	RR=1.5	0.61,3.69	72.1	-1	-1	0	-1	0	Critically low
	Tofacitinib I0 mg bid	RR=2.75	1.72,4.41	0	-1	0	0	0	0	Medium
	Baritnib 2 mg qd	RR=1.06	0.72,1.57	67.2	-1	-1	0	-1	0	Critically low
	Baritinib 4 mg qd	RR=1.28	1.I 2,1.45	0	-1	0	0	0	0	Medium
	Upadacitinib 15 mg qd	RR=1.35	1.14,1.60	24.4	-1	0	0	0	0	Medium
	Upadacitinib 30 mg qd	RR=1.28	0.96,l.70	39.3	-1	0	0	-1	0	Low
	**Serious infections**									
	Tofacitinib 5 mg bid	RR=2.08	0.43,10.02	0	-1	0	0	-2	0	Critically low
	Tofacitinib I0 mg bid	RR=2.26	0.64,8.01	0	-1	0	0	-2	0	Critically low
	Barictinib 2 mg qd	RR=0.97	0.28,3.37	30	-1	0	0	-1	0	Low
	Baitinib 4 mg qd	RR=0.94	0.47,1.90	30	-1	0	0	-1	0	Low
	Upadaitinib 15 mg qd	RR=2.23	0.88,5.64	0	-1	0	0	-1	0	Low
	Upadacitinib 30 mg qd	RR=4.59	0.77,27.34	0	-1	0	0	-2	0	Critically low

			**Herpes zoster**									
			Tofacitinib 5 mg bid	RR=1.66	0.21,13.28	0	-1	0	0	-1	0	Low
			Tofacitinib I0 mg bid	RR=6.94	0.89,54.06	0	-1	0	0	-1	0	Low
			Baritinib 2 mg qd	RR=2.32	0.27,19.93	37.4	-1	0	0	-1	0	Low
			Baritinib 4 mg qd	RR=3.81	1.35,10.71	0	-1	0	0	-2	0	Critically low
			Upadaitinib I5 mg qd	RR=1.41	0.44,4.45	0	-1	0	0	-1	0	Low
			Upadaitinib 30 mg qd	RR=2.96	0.59,14.83	0	-1	0	0	-2	0	Critically low
			**Upper respiratory tract infection**									
			Tofacitinib 5 mg bid	RR=1.61	1.00,2.59	0	-1	0	0	0	0	Medium
			Tofacitinib 10 mg bid	RR=1.6	0.83,3.06	28.7	-1	0	0	-1	0	Low
			Baritinib 2 mg qd	RR=1.21	0.47,3.11	68.1	-1	-1	0	-1	0	Critically low
			Baritinib 4 mg qd	RR=1.31	0.80,2.14	0	-1	0	0	-1	0	Low
			Upadaitinib I5 mg qd	RR=1.15	0.66,2.01	0	-1	0	0	-1	0	Low
			Upadacitinib 30 mg qd	RR=1.01	0.57,1.81	0	-1	0	0	-1	0	Low
												+++-
**Wang**	2022	37/15174	**ACR20**									
			All RCTs	RR=2.03	1.85,2.23	65	-1	-1	0	0	0	Low
			Tofacitinib	RR=2.21	1.86,2.63	52	-1	0	0	0	0	Medium
			Barictinib	RR=1.95	1.57,2.42	78	-1	-1	0	0	0	Low
			Upadacitinib	RR=1.99	1.68,2.36	64	-1	-1	0	0	0	Low
			Decernotinib	RR=2.61	1.70,4.01	31	-1	0	0	0	0	Medium
			Peficinib	RR=2.01	1.32,3.05	84	-1	-1	0	0	0	Low
			Filgotinib	RR=1.8	1.43,2.27	46	-1	0	0	0	0	Medium
			**ACR50**		,							
			All RCTs	RR=3.12	2.48,3.93	84	-1	-1	0	0	0	Low

Tofacitinib	RR=3.43	2.30,5.12	78	-1	-1	0	0	0	Low
Barictinib	RR=2.73	2.03,3.66	0.64	-1	0	0	0	0	Medium
Upadacitinib	RR=2.25	1,12,4.52	0.96	-1	0	0	0	0	Medium
Decernotinib	RR=4.72	2.48,8.96	0	-1	0	0	0	0	Medium
Peficinib	RR=2.84	1.42,5.70	0.82	-1	0	0	0	0	Medium
Filgotinib	RR=5.56	2.79,11.06	0.11	-1	0	0	0	0	Medium
**ACR70**									
All RCTs	RR=3.87	3.02,4.97	56	-1	-1	0	0	0	Low
Tofacitinib	RR=4.15	2.21,7.80	74	-1	-1	0	0	0	Low
Barictinib	RR=3.81	2.97,4.89	0	-1	0	0	0	0	Medium
Upadacitinib	RR=4.53	3.53,5.83	0	-1	0	0	0	0	Medium
Decernotinib	RR=4.06	1.50,10.98	0	-1	0	0	0	0	Medium
Peficinib	RR=3.64	1.32,10.05	73	-1	-1	0	0	0	Low
Filgotinib	RR=3.41	0.94,12.40	45	-1	-1	0	-2	0	Critically low
**HAQ-DI**									
All RCTs	RR=-0.31	-0.34,-0.28	0	-1	0	0	0	0	Medium
Tofacitinib	RR=-0.34	-0.39,-0.28	0	-1	0	0	0	0	Medium
Barictinib	RR=-0.24	-0.33,-0.15	0	-1	0	0	0	0	Medium
Upadacitinib	RR=-0.31	-0.36,0.26	0	-1	0	0	0	0	Medium
Decernotinib	RR=-0.24	-0.48,-0.01	0.72	-1	0	0	0	0	Medium
Peficinib	RR=-0.22	-0.42,-0.02	-,	-1	-1	0	0	0	Low
Filgotinib	RR=-0.33	-0.44,-0.22	0.44	-1	0	0	0	0	Medium
**Adverse events**									
All RCTs	RR=1.1	1,05,1.14	0.25	-1	0	0	0	0	Medium
Tofacitinib	RR=1.06	0.98,1.15	0.29	-1	0	0	-1	0	Low
Barictinib	RR=1.1	1,01,1.21	0.48	-1	0	0	0	0	Medium

	Upadacitinib	RR=1.19	1,11,1.28	0.07	-1	0	0	0	0	Medium
	Decernotinib	RR=1.32	0.97,1.78	0.4	-1	0	0	-1	0	Low
	Peficinib	RR=1.04	0.94,1.16	0	-1	0	0	-1	0	Low
	Filgotinib	RR=0.96	0.84,1.10	0	-1	0	0	-1	0	Low
	**Serious Adverse events**									
	All RCTs	RR=0.94	0.77,1.15	0	-1	0	0	-1	0	Low
	Tofacitinib	RR=0.74	0.47,1.18	0.2	-1	0	0	-1	0	Low
	Barictinib	RR=0.92	0.65,131	0	-1	0	0	-1	0	Low
	Upadacitinib	RR=1.72	0.92,325	0.18	-1	0	0	-1	0	Low
	Decernotinib	RR=1.47	0.58,3.71	0	-1	0	0	-1	0	Low
	Peficinib	RR=0.95	0.46,1.96	0	-1	0	0	-1	0	Low
	Filgotinib	RR=0.7	0.24,2.07	0.46	-1	0	0	-1	0	Low
	**Thromboembolic events**									
	All RCTs	RR=1.04	0.38,2.84	0	-1	0	0	-1	0	Low
	Tofacitinib	RR=0.19	0.01,2.91	0.35	-1	0	0	-1	0	Low
	Barictinib	RR=2.38	0.27,20.84	0	-1	0	0	-2	0	Critically low
	Upadacitinib	RR=1.65	0.33,8.35	0	-1	0	0	-1	0	Low
	Decernotinib	RR=0.77	0.03,18.52	NA	-1	-1	0	-2	0	Critically low
	Peficinib	NA	NA	NA	-1	-1	0	-2	0	Critically low
	Filgotinib	RR=1.49	0.06,36.24	NA	-1	-1	0	-2	0	Critically low
	**Neoplasms**									
	All RCTs	RR=1.7	0.74,3.89	0	-1	0	0	-1	0	Low
	Tofacitinib	RR=9.5	0.56,162.20	NA	-1	-1	0	-2	0	Critically low
	Baricitinib	RR=1.03	0.26,4.10	0	-1	0	0	-1	0	Low
	Upadacitinib	RR=1.5	0.40,5.54	0	-1	0	0	-1	0	Low

Decernotinib	RR=2.92	0.35,24.20	0	-1	0	0	-2	0	Critically low
Peficinib	NA	NA	NA	-1	-1	0	-2	0	Critically low
Filgotinib	NA	NA	NA	-1	-1	0	-2	0	Critically low
**Infections**									
All RCTs	RR=1.29	1.19,1.39	0	-1	0	0	0	0	Medium
Tofacitinib	RR=1.3	1.00,1.94	0	-1	0	0	0	0	Medium
Barictinib	RR=1.22	1.09,1.37	0	-1	0	0	0	0	Medium
Upadacitinib	RR=1.38	1.22,1.56	0	-1	0	0	0	0	Medium
Decernotinib	RR=1.43	0.80,2.58	37	-1	0	0	-1	0	Low
Peficinib	RR=1.01	0.66,1.56	0	-1	0	0	-1	0	Low
Filgotinib	RR=1.5	0.53,4.20	37	-1	0	0	-1	0	Low
**Serious Infections**									
All RCTs	RR=1.3	0.92,1.86	0	-1	0	0	-1	0	Low
Tofacitinib	RR=1.35	0.72,2.55	0	-1	0	0	-1	0	Low
Barictinib	RR=0.91	0.48,1.71	0	-1	0	0	-1	0	Low
Upadacitinib	RR=1.92	0.83,4,47	4	-1	0	0	-1	0	Low
Decernotinib	RR=2.58	0.49,13.63	0	-1	0	0	-1	0	Low
Peficinib	RR=2.63	0.59,11.73	0	-1	0	0	-1	0	Low
Filgotinib	RR=0.67	0.18,2.44	0	-1	0	0	-1	0	Low
**Herpes zoster**									
All RCTs	RR=1.59	1.09,2.32	0	-1	0	0	0	0	Medium
Tofacitinib	RR=1.28	0.72,2.29	0	-1	0	0	-1	0	Low
Barictinib	RR=3.15	1.19,8.33	0	-1	0	0	0	0	Medium
Upadacitinib	RR=1.25	0.56,2.81	0	-1	0	0	-1	0	Low
Decernotinib	RR=1.79	0.09,34.04	NA	-1	-1	0	-2	0	Critically low
Peficinib	RR=2.13	0.51,8.92	37	-1	0	0	-1	0	Low

			Filgotinib	RR=0.97	0.21,4.51	0	-1	0	0	-1	0	Low
			**Upper respiratory infection**									
			All RCTs	RR=1.26	0.97,1.63	0	-1	0	0	-1	0	Low
			Tofacitinib	RR=1.2	0.69,2.10	33	-1	0	0	-1	0	Low
			Barictinib	RR=1.22	0.78,1.89	0	-1	0	0	-1	0	Low
			Upadacitinib	RR=1.34	0.63,2.83	NA	-1	-1	0	-1	0	Critically low
			Decernotinib	RR=1.24	0.28,5.52	NA	-1	-1	0	-1	0	Critically low
			Peficinib	RR=1.6	0.69,3.67	0	-1	0	0	-1	0	Low
			Filgotinib	RR=0.89	0.30,2.60	NA	-1	-1	0	-1	0	Critically low
			**MACE**									
			All RCTs	RR=1.02	0.45,2.34	0	-1	0	0	-1	0	Low
			Tofacitinib	RR=2.43	0.31,19.07	0	-1	0	0	-2	0	Critically low
			Barictinib	RR=0.59	0.10,3.40	21	-1	0	0	-1	0	Low
			Upadacitinib	RR=1.17	0.32,4.22	0	-1	0	0	-1	0	Low
			Decernotinib	RR=0.76	0.08,7.22	0	-1	0	0	-1	0	Low
			Peficinib	NA	NA	NA	-1	-1	0	-2	0	Critically low
			Filgotinib	NA	NA	NA	-1	-1	0	-1	0	Critically low
**He**	2013	8/3791	**ACR20(12 weeks)**									
			Tofacitinib 1mg vs.placebo	RR=1.83	1.00,3.32	63	-1	-1	0	0	0	Low
			Tofacitinib 3mg vs.placebo	RR=2.20	1.20,4.04	67	-1	0	0	0	0	Medium
			Tofacitinib 5mg vs.placebo	RR=2.20	1.58,3.07	64	-1	0	0	0	0	Medium
			Tofacitinib 10mg vs.placebo	RR=2.38	1.81,3.14	51	-1	-1	0	0	0	Low
			Tofacitinib 15mg vs.placebo	RR=2.29	1.19,4.41	76	-1	0	0	0	0	Medium
			**ACR20(24 weeks)**									
			Tofacitinib 5mg vs.placebo	RR=1.94	1.55,2.44	0	-1	0	0	0	0	Medium

Tofacitinib 10mg vs.placebo	RR=2.20	1.76,2.75	0	-1	0	0	0	0	Medium
**ACR50(12 weeks)**									
Tofacitinib 5mg vs.placebo	RR=2.91	2.03,4.16	0	-1	0	0	0	0	Medium
Tofacitinib 10mg vs.placebo	RR=3.32	2.33,4.72	0	-1	0	0	0	0	Medium
**ACR50(24 weeks)**									
Tofacitinib 5mg vs.placebo	RR=3.61	2.08,6.26	0	-1	0	0	0	0	Medium
Tofacitinib 10mg vs.placebo	RR=4.73	2.76,8.09	0	-1	0	0	0	0	Medium
Infection 5mg bid									
Upper respiratory tract infection	RR=1.12	0.61,2.05	Not Mentioned	-1	-1	0	-1	0	Critically low
Urinary tract infection	RR=1.22	0.58,2.57	Not Mentioned	-1	-1	0	-1	0	Critically low
Bronchitis	RR=0.80	0.25,2.56	Not Mentioned	-1	-1	0	-1	0	Critically low
Nasopharyngitis	RR=1.57	0.51,4.83	Not Mentioned	-1	-1	0	-1	0	Critically low
Influenza	RR=0.37	0.08,1.64	Not Mentioned	-1	-1	0	-1	0	Critically low
Pharyngitis	RR=0.01	-0.01,0.03	Not Mentioned	-1	-1	0	0	0	Low
Immune system									
Neutropenia	RR=1.41	0.55,3.61	Not Mentioned	-1	-1	0	-1	0	Critically low
Rash	RR=0.32	0.04,2.61	Not Mentioned	-1	-1	0	-1	0	Critically low
Infection 10mg bid									

			Upper respiratory tract infection	RR=0.77	0.40,1.49	Not Mentioned	-1	-1	0	-1	0	Critically low
			Urinary tract infection	RR=1.01	0.45,2.27	Not Mentioned	-1	-1	0	-1	0	Critically low
			Bronchitis	RR=1.01	0.34,2.96	Not Mentioned	-1	-1	0	-1	0	Critically low
			Nasopharyngitis	RR=1.67	0.82,3.39	Not Mentioned	-1	-1	0	-1	0	Critically low
			Influenza	RR=1.25	0.25,6.20	Not Mentioned	-1	-1	0	-1	0	Critically low
			Pharyngitis	RR=0.03	-0.09,0.14	Not Mentioned	-1	-1	0	0	0	Low
			Immune system									
			Neutropenia	RR=1.73	0.68,4.38	Not Mentioned	-1	-1	0	-1	0	Critically low
			Rash	RR=2.51	0.63,9.93	Not Mentioned	-1	-1	0	-1	0	Critically low
			Withdrawal									
			All causes	RR=0.60	0.45,0.78	Not Mentioned	-1	-1	0	-1	0	Critically low
			Adverse events	RR=1.43	0.68,3.03	Not Mentioned	-1	-1	0	-1	0	Critically low
			Lack of efficacy	RR=0.18	0.09,0.35	Not Mentioned	-1	-1	0	0	0	Low
**Kawale**	2013	8/-	ACR20(12 weeks)									

**c**										
	Tofacitinib+MTX vs.placebo+MTX	RR=0.48	0.36,0.64	61	-1	-1	0	0	-1	Critically low
	Tofacitinib vs.placebo	RR=0.35	0.23,0.54	56	-1	-1	0	0	-1	Critically low
	ACR50(12 weeks)									
	Tofacitinib vs.Adalimumab	RR=0.65	0.49,0.87	0	-1	0	0	-1	-1	Critically low
	AE(12 weeks)									
	Tofacitinib+MTX vs.placebo+MTX	RR=1.22	0.47,3.20	34	-1	-1	0	-1	-1	Critically low
	Tofacitinib vs.placebo	RR=0.33	0.31,2.14	49	-1	-1	0	-1	-1	Critically low
